# Noninvasive Brain Stimulation Techniques and Their Efficacy in Treating Cognition and Memory in Mild Cognitive Impairment and Alzheimer’s Disease—A Systematic Review

**DOI:** 10.3390/brainsci16050527

**Published:** 2026-05-15

**Authors:** Hector P. Valverde, Benjamin J. Clark, Jeremy Hogeveen, Vincent P. Clark

**Affiliations:** 1 Psychology Clinical Neuroscience Center, Department of Psychology, University of New Mexico, Albuquerque, NM 87131, USA; hpvalverde@unm.edu (H.P.V.); bnjclark@unm.edu (B.J.C.); jhogeveen@unm.edu (J.H.); 2 Energy Creating Arts, Albuquerque, NM 87122, USA

**Keywords:** neuromodulation, transcranial direct current stimulation, transcranial alternating current stimulation, transcranial focused ultrasound stimulation, transcranial magnetic stimulation, dementia, Alzheimer’s disease, noninvasive brain stimulation

## Abstract

**Highlights:**

**What are the main findings?**

- Across 81 studies, noninvasive brain stimulation showed promise for improving cognition and memory in amnestic mild cognitive impairment (aMCI) and Alzheimer’s disease.
- Transcranial magnetic stimulation (TMS) showed the most durable benefits, while transcranial electrical stimulation (TES) effects were often shorter-lived but with fewer side effects, and transcranial focused ultrasound stimulation (tFUS) remains preliminary but promising.

**What are the implications of the main findings?**

- Early, repeated, and network-targeted stimulation may be most useful for treating cognitive symptoms in prodromal and Alzheimer’s disease populations.
- Future studies should use more standardized protocols, longer follow-up, and stronger biomarker validation to define clinical efficacy.

**Abstract:**

Background/Objectives: The growing aging population is susceptible to cognitive and memory impairment, most commonly due to Alzheimer’s disease, with no cures currently available. Noninvasive brain stimulation (NIBS) techniques may serve to improve cognition and delay catastrophic memory loss. Methods: A systematic review of NIBS research on cognitive impairment was carried out using PubMed, with additional backward citation searching. A total of 81 studies using NIBS were included. Conclusions: The reviewed studies show that NIBS holds promise in improving memory deficits in patients with cognitive impairment. While the longevity of benefits from transcranial electrical stimulation appears limited, its short-term effects may provide benefits when used consistently. Transcranial magnetic stimulation appears to provide longer-lasting benefits. Transcranial focused ultrasound stimulation may also provide further benefits through more precise targeting of deeper brain structures compared to other NIBS techniques. Together, these results suggest that NIBS shows promise for the treatment of symptoms related to cognitive and memory impairment, and may help to alleviate some of the growing issues associated with the increasing level of Alzheimer’s disease in an aging population.

## 1. Introduction

Dementia poses one of the biggest, most debilitating threats to human health and quality of life in older adults today. Its afflictions involve the gradual loss of cognitive abilities including memory, language, and executive function, most commonly due to a combination of cerebrovascular and neurodegenerative diseases [1,2]. The most prevalent form of neurodegenerative dementia is Alzheimer’s disease (AD), whose distinct pathological profile progressively impairs individuals’ ability to acquire and retain memories, and eventually their capacities for reasoning and language, as well [3]. The greatest risk factor for AD is advancing age, and the disease’s global impact is believed to continue to rise in the future as the proportion of older adults in populations around the world is only growing due to increasing life expectancy [4]. There is currently no cure for AD, and a method to prevent the progression of the disease safely and cheaply has proven elusive. Anti-amyloid monoclonal antibody drugs are a relatively recent innovation attempting to treat the underlying pathology of the disease. These are promising and have shown the ability to slow disease progression, albeit with high cost, small effect sizes and significant potential to induce dangerous side effects such as brain hemorrhages [5]. It is vital to develop better biomarkers for patients in early, prodromal stages of dementia—to drive the development of preventative interventions that could slow or reverse the typical progression symptoms before they advance.

Synaptic loss and dysfunction correlate highly with reduced performance on cognitive tests [6], and precede many forms of subsequent neurodegeneration associated with the pathophysiology of dementia [3]. Mild cognitive impairment (MCI), particularly the amnestic variant (aMCI), has been flagged as a potential prodromal stage of AD due to its similar expressions of pathology alongside cognitive deficits [7,8,9], with synaptic degeneration being observed in this early stage [10,11]. As such, alongside drugs that target pathology in attempts to stop progression of the disease, many of the pharmacological treatments presently dominating clinical trials research focus on the recalibration of altered, AD-related synaptic neurotransmission to ameliorate behavioral and cognitive symptoms. Although medications such as acetylcholinesterase inhibitors (AChEIs) and N-methyl-d-aspartate receptor (NMDAR) antagonists have been successful in reducing symptoms of dementia, their efficacy has proven mild, variable, and/or transient at best, along with producing a number of unpleasant side effects [12,13].

Finding next-generation treatment approaches for treating aMCI and early stages of AD is a major public health challenge. One new approach—noninvasive brain stimulation (NIBS)—has recently emerged as a novel approach to treating aMCI and AD with minimal side effects, a demonstrated efficacy to influence synaptic plasticity, and effects outlasting an immediate stimulation session in increments of days, weeks, or months. NIBS may therefore provide a safer and more effective treatment strategy in these patient populations.

The purpose of this document is to provide a general summary of current findings pertaining to the use of NIBS in aMCI and AD populations. To accomplish this, the mechanisms of action of some of the most frequently used and promising NIBS techniques will be detailed, including transcranial electrical stimulation (TES), transcranial magnetic stimulation (TMS), and transcranial focused ultrasound stimulation (tFUS). Then, studies on these NIBS modalities with aMCI and AD patients will be reviewed and discussed with potential methods and concerns that could be used to direct future research.

## 2. Mechanisms of Action of Noninvasive Brain Stimulation Techniques

### 2.1. Synaptic Plasticity

The primary mechanism of action for NIBS-based interventions is their ability to induce lasting changes in synaptic plasticity in targeted circuits. Synaptic plasticity refers to activity-dependent changes in synaptic transmission efficacy. Principles of Hebbian plasticity state that acute, repeated communication between neurons will promote synaptic connectivity while less frequent interaction will lead to synaptic decay [14]. Bliss and Lømo’s foundational experiment on anesthetized rabbit brains yielded convincing evidence for this theory as manual, high-frequency electrical stimulation of the perforant path yielded long-lasting changes to synaptic transmission within the region [15]. Subsequent research has deemed NMDAR-dependent signaling between neurons to be the principal driver of more enduring synaptic modifications known as long-term potentiation (LTP) and long-term depression (LTD) [16,17], and further rodent research has associated long-term synaptic changes with processes pertaining to memory through more naturalistic paradigms of learning and experience [18].

It is believed that the strength and duration of the neuromodulatory effects of NIBS acutely operate on similar concepts. NIBS studies in animal models have corroborated this theory as increased expression of markers of synaptic plasticity such as brain-derived neurotrophic factor (BDNF), cyclic adenosine monophosphate-response element binding protein (CREB), and calmodulin-dependent protein kinase II (CaMKII) have been found in the dissected hippocampi of healthy rats after receiving TES, TMS, or tFUS [19,20,21]. Notably, in tFUS, this change has been observed within a window as short as only 40 s of stimulation [21]. Similar benefits have also been extended to animal models of AD [22], with the additional benefits of clearance of pathology and cognitive improvement being observed when sufficient intensity is used [22,23,24].

Neurons not consistently integrated into a wider network response pattern or that fire at less coordinated times also hold the potential to diminish the overall degree of networked LTP over time while inducing their own LTD [25]. In this regard, the course of LTP and LTD can be influenced by cross-neuronal interactions related to spike-timing-dependent plasticity (STDP). STDP refers to the temporal window in which pre- and post-synaptic activity interacts across multiple neurons; pre-to-post-synaptic interactions have been found to induce LTP changes through facilitatory activation that may back-propagate into repeated cell-to-cell firing while post-to-pre-synaptic interactions are prone to developing LTD due to unsynchronized or unassociated activity between neurons [16]. In this regard, although LTP is dependent on repeated excitatory activity, neuronal communication in the short term also involves innate refractory periods following the release of an action potential. Vesicular reuptake and other homeostatic resets in this period may be interrupted if consecutive trains of excitatory activity take place within 20 msec of each other, causing a neuron to depress a potential response, while trains with an interval of 20–500 msec establish the optimal post-recovery window for a neuron to fire, potentially facilitating synaptic reaction to a specific stimulus multiple times and across multiple neighboring neurons, as with STDP-induced LTP [26]. As aMCI/AD pathology fosters excitotoxic environments, the development of LTD and suppression of LTP over time may be due to these desynchronized neuronal interactions. Additionally, it may also be reasonable to theorize that unattenuated NIBS not tuned properly to endogenous activity may result in similar depressions of activity or, at the very least, less pronounced effects from excitation.

These general principles of plasticity are likely further constrained in aMCI and AD by disease-specific pathology. Amyloid- and tau-related synaptic dysfunction, reductions in neurotrophic support, and abnormalities in intracellular signaling linked to BDNF/CREB- and phosphatidylinositol 3-kinase (PI3K)/Akt/glycogen synthase kinase 3 beta (GSK-3β)-related cascades may all reduce the efficiency with which hippocampal–cortical circuits support memory formation and consolidation [3,12,13,27]. Accordingly, the therapeutic rationale for NIBS in dementia is not simply that stimulation can transiently alter excitability, but that appropriately timed and targeted stimulation may partially restore more favorable conditions for network synchronization, plasticity, and mnemonic processing in circuits destabilized early in the disease course.

### 2.2. Transcranial Electrical Stimulation (TES)

TES typically uses two (or sometimes more) electrodes filled with a conductive media (gel or saline) to pass a weak electrical current through the scalp and skull into the brain in an effort to influence the threshold of local neuronal excitability [28,29]. Approximately 1–2 mA is typically delivered in TES studies [30,31], with recommended safety parameters setting a limit of up to 4 mA of electrical current. After passing through the scalp and skull, only about 10–25% of that current is able to reach the cortex [31]. As such, this form of stimulation does not necessarily affect neuronal activity by initiating or silencing action potentials; rather, the small electrical current is thought to slightly sway the resting membrane potential of neurons to increase or decrease the likelihood that their firing threshold will be crossed [28,32]. Specifically, it is believed that the electrical field that reaches the brain affects the spontaneous firing rates of neurons by acting on pre-synaptic calcium and sodium voltage-gated ion channels, and by conditioning synaptic activity and neurotransmitter release through repeated activation or suppression, can lead to the downstream modulation of post-synaptic NMDAR efficacy even after stimulation has ceased [27,29].

The low cost, compact size, and ease of administering TES make it an excellent candidate for use both in clinics and at home. Furthermore, TES is considered a safe method of brain stimulation as the typical current density being delivered is fractionally several thousand times below the threshold to damage tissue and several tens of thousands of times below the ability to cause a brain lesion [30]. A review on the reported adverse effects of TES by Antal et al. [33] found that typical adverse effects include itching, burning or tingling sensations, headaches and mild pain, as well as the presentation of retinal phosphenes at certain frequencies. In the over 18,000 sessions surveyed across over 8000 subjects (including both healthy and clinical populations), these adverse effects ranged from minor to moderate but were considered tolerable and transient, and the few serious adverse effects reported were not directly associated with TES administration.

Another consideration in the use of TES is the orientation of neurons relative to the electrical field being applied [27,28,31,33] and thus how electrode montages are modeled to stimulate their target. As a result, it is highly probable that interindividual differences within research studies, such as differences in skull conductivity, brain anatomy and cerebrospinal fluid volume, may affect the efficacy of TES. Lack of replication and consistency is a known problem across TES literature [34], of which a large amount has been attributed to uncontrolled heterogenous characteristics among research subjects that may diminish the effectiveness of stimulation parameters. Modeling work done by Opitz et al. [35] attributed up to 50% of the variance in electrical field strength and distribution at a targeted brain region to anatomical factors involving the skull, brain, and cerebrospinal fluid layers, as well as the distance and orientation of the electrodes relative to each other. A recent review by Vergallito et al. [36] further honed in on three defining sources of interindividual variability in studies using TES: stable factors (including demographic features such as gender and age, as well as anatomical features in skull thickness and brain morphology); variable state-based factors (hormones and influential exogenous substances such as medications, caffeine, and the like); and experimental contextual elements (state dependency between task and stimulated brain regions, as well as baseline capabilities of participants). Although there is generally no way to control for anatomical factors, exclusionary or restrictive criteria for participant conditions within testing conditions, as well as experimental design, are critical in stabilizing variability of the results across TES studies.

In terms of temporal characteristics of TES, two of the most studied methods are transcranial direct current stimulation (tDCS) and transcranial alternating current stimulation (tACS). For the purposes of this document, only tDCS and tACS will be described further as a very limited number of research studies using other forms of TES in aMCI and AD populations have been conducted. Although the methods of delivery in tDCS and tACS are similar, the ways in which they are thought to modulate neuronal activity are quite different. In terms of spatial characteristics, TES is intended to modulate the raw firing rate of neurons. Electrical current is introduced into the targeted area by flowing from one or more negatively charged cathode electrodes to one or more positively charged anode electrodes [37]. Electrode placement thus determines the effects of stimulation in tDCS protocols: cathodal stimulation (in which the cathode is placed over the target) reduces neuronal activity, whereas anodal stimulation (anode is placed over the target) excites activity [28,29]. The use of large, rectangular electrode montages leads to greater field strength around the edges of the electrodes relative to the center [38]. Improved focality and penetration depth have been achieved with High-Definition tDCS (HD-tDCS), a specialized form of tDCS whose most common montage, 4 × 1 HD-tDCS, places four small circular ring electrodes surrounding the region of interest, with a fifth electrode of opposite polarity over the target region [39]. For excitatory stimulation, the four surrounding electrodes are cathodal while the fifth electrode is anodal [38].

In contrast to tDCS, current flow in tACS alternates between multiple electrodes, usually in a sinusoidal pattern, in an effort to entrain endogenous cortical oscillations to an applied electrical frequency [40,41]. In other words, tACS acts by modulating the timing of neuronal firing en masse. Greater populations of neurons and interacting networks can be recruited when a tACS frequency closely matches ongoing intrinsic oscillations (a state known as “resonance”), so in addition to entraining a close phase alignment between the two waveforms, task relevance and the frequency of stimulation must be carefully tuned together to capitalize on this method’s effects [42]. The inverse can also be true; Polanía et al. [43] elegantly demonstrated the relationship between resonance and phase alignment between exogenous and endogenously applied frequencies through a series of experiments using a delayed letter recognition task. After EEG analyses determined that the task was most associated with 6 Hz endogenous activity involving the left prefrontal and parietal cortices, sham, in-phase, and out-of-phase 6 Hz theta tACS was applied over the dorsolateral prefrontal cortex and posterior parietal cortex. Out-of-phase 6 Hz tACS resulted in slower reaction times compared to sham while in-phase stimulation saw faster reaction times. Comparatively, when hypothetically non-resonant 35 Hz gamma tACS was applied using the same task, reaction times were similar across the three stimulation conditions.

### 2.3. Transcranial Magnetic Stimulation (TMS)

TMS involves the rapid discharge of high-voltage electrical current from a magnetic stimulating coil of insulated copper wire to produce a magnetic field capable of penetrating the skull and generating an electrical field within brain tissue [44,45,46]. These generated fields typically last for approximately 100 µs, referred to as pulses [47] and, like the mechanisms of TES, are believed to alter neuronal excitability through the electrical modulation of voltage-gated ion channels, such as NMDARs [44,45]. By manifesting directly within cortical tissue, however, TMS circumvents the shunting effects encountered in TES, and the produced electrical currents are able to induce action potentials on their own for potentially more pronounced neuromodulatory effects [32]. In comparison to a traditional TES montage, TMS additionally provides increased stimulation focality, decreasing the potential for noise to be introduced when targeting specific brain regions. The produced magnetic and electrical fields can further vary based on the orientation and the shape of the coil used, trading increased focality for decreased penetration. For example, figure-eight coils typically provide better field focality compared to circular loop designs and can improve it by as much as 29 cm^2^, albeit featuring a tradeoff of less than a centimeter of penetration depth from the surface of the cortex [48]. On average, the capacity for TMS using a typical coil to penetrate the cortex ranges between 1.5 and 3 cm beneath the scalp [45].

While single pulse and paired pulse TMS paradigms have largely been used as diagnostic tools to assess neuronal and network conductivity thresholds across different brain areas and clinical populations [46], long-lasting modulation of neuronal activity has been accomplished by applying patterned trains of multiple stimulation pulses in varying timed intervals, a technique known as repetitive TMS (rTMS). With all other variables held constant, low-frequency rTMS (≤1 Hz) has been found to inhibit cortical excitability [49] and high-frequency rTMS (>5 Hz) facilitates excitability [50]. Alongside the frequency used, one of the main determinants of the effects and longevity of rTMS is the stimulation intensity applied. Stimulation intensity is most commonly based on motor thresholds (MTs) [51], which is the minimum TMS machine output needed to elicit a motor-evoked potential of at least 50 µV in at least half of a series of pre-determined trials for a given individual [52]. Although this is generally accepted as a benchmark of stimulation intensity across TMS research, potential discrepancies between the excitability of brain regions respective to the motor cortex have been acknowledged [45]. A review by Turi et al. [51] found the majority of rTMS research uses responses to relaxed muscles, or resting motor thresholds (rMTs), in comparison to active motor thresholds (aMTs), in which muscles are voluntarily contracted.

Theta burst stimulation (TBS) protocols are variations of rTMS that apply bursts of three pulses at 50 Hz, and intermittent TBS (iTBS) protocols, in which two-second trains are applied every 10 s over a total of 600 pulses, have produced stronger and longer-lasting excitatory effects relative to other high-frequency rTMS methods [53]. Reciprocally, the same research has found continuous TBS (cTBS), in which trains are applied for 20 s, to produce stronger inhibitory effects [53]. An overall benefit of TBS is its accelerated stimulation delivery, which can create treatment sessions as short as 3 min in comparison to the 20–30 min of other protocols. In addition to facilitating tolerability for clinical populations with decreased stimulation times, this delivery also allows for multiple sessions of stimulation in a single clinical visit, consolidating treatment schedules considerably.

The use of stereotaxic navigation systems is common in TMS research, which can assist in creating accurate and repeatable stimulation parameters while reducing some potential for intraindividual variability across multiple stimulation sessions [54]. Regardless, a review by Pell et al. [55] finds several sources for response variability to TMS treatment, notably conditions affecting geometric interactions between produced electrical fields and neurons (coil positions relative to cortical gyri, accurate and representative functional magnetic resonance imaging (fMRI) and stereotaxic registration); timing-based factors such as the relationship between stimulation frequency and interstimulus intervals, per theory of STDP; and other factors including demographics (gender, age, hormones), interindividual morphological variability, and state dependence (exogenous substances, alertness/excitability/attention, cognitive state). There is also increasing evidence that certain frequencies within the spectrum of high-frequency rTMS may be more effective than others at producing local neuromodulatory effects due to potential entrainment and facilitation of resonant endogenous brain oscillations [56]. Although these entrainment effects appear to scale positively with stimulation intensity, they have also been produced with electrical field strengths significantly below established MT values [57], suggesting relevant cortical regions and/or networks may be more susceptible to being influenced by similarly oriented exogenous stimulation [58]. Additionally, alongside stimulation frequency and intensity, a balance between pulse quantity and number of stimulation sessions has also been flagged in mechanisms mediating efficacy of rTMS, with findings suggesting that multiple sessions may be more effective at producing neuromodulatory effects relative to a raw number of stimulation pulses, which may reach ceiling effects within an individual treatment session [59].

rTMS and TBS are generally tolerated well by most patients in clinical research despite some documented negative side effects. Per Rossi et al. [45], the most serious potential side effects, induction of seizures and scalp burns, are rare (seizures were found limited to 1.4% in epileptics and less than 1% in non-epileptics) when the recommended safety guidelines are followed, and more frequently reported side effects such as headaches, bodily pains, and cognitive changes have been found transient. In a follow-up review, Rossi et al. [60] concluded that the previously established dosing parameters balancing TMS frequency, intensity, train durations, pulse quantity, interstimulus intervals, and scheduling of stimulation sessions have held up well, with no consistent upticks in negative side effects or reported seizures despite significant increases in TMS research over the past twelve years. For a more thorough review of the documented side effects and current safety parameters for TMS protocols, see Rossi et al. [45,60].

A related line of work that has largely been conducted in cognitively normal younger or older adults rather than in aMCI/AD cohorts and therefore is outside the formal scope of the present review is highly relevant here. Across a series of individualized resting-state fMRI-guided TMS studies, stimulation targets were selected in the lateral parietal or posterior-medial cortex based on functional connectivity with the hippocampus, and these approaches were shown to increase hippocampal-network connectivity, with several studies also demonstrating selective improvements in associative, episodic, or recollection-based memory performance [61,62,63,64,65,66]. These findings are important for dementia research because they provide mechanistic support for the idea that precision targeting of cortical nodes within hippocampal–cortical networks can modulate hippocampal-dependent memory operations. Future applications of network-guided TMS approaches may result in similar benefits in aMCI and AD populations.

### 2.4. Transcranial Focused Ultrasound Stimulation (tFUS)

tFUS is a relatively new and developing noninvasive brain stimulation technique that employs beams of ultrasonic sound waves to modulate neuronal activity. At frequencies of 200–900 kHz, the acoustic energy transmitted from a piezoelectric transducer has the ability to pass through cortical tissue and the skull [67] to reach deep brain structures previously inaccessible to other NIBS techniques.

Though ultrasound technology has been studied and used in practice for the greater part of a century, the field of neuromodulatory tFUS is still relatively new, and as such, the specifics behind its mechanisms of action are currently unclear. One of the leading theories suggests that the acoustic pressure induced by tFUS may stretch or displace phospholipid membranes. Accordingly, these physical changes may open or close the mechano-sensitive ion channels embedded within them, causing downstream shifts in membrane potentials and neuronal activity [68,69]. A study by Prieto et al. [70] found potassium (K+) channels may be specifically associated with the excitatory effects of tFUS as forced K+ influx induced by stimulation allowed for rapid repolarization of neurons immediately following the release of an action potential, theoretically facilitating subsequent activity and inciting LTP-like effects. The authors additionally found K+ channels were particularly susceptible to the subtle heating effects of low-intensity tFUS, suggesting an adjacent thermo-sensitive explanation for the mechanisms of tFUS neuromodulation alongside the mechano-sensitive alternative. One other theory posits that the aforementioned distortion of the phospholipid bilayer can displace current along the membrane and influence proximal voltage-gated ion channels, eliciting a greater overall change in conductance across the membrane and subsequent buildup to depolarization [69].

The stimulation parameters determining the effects of tFUS include fundamental frequency (FF), sonication duration (SD), pulse repetition frequency (PRF), duty cycle (DC), and intensity [71]. Fundamental frequency (typically measured in kHz) is the chosen frequency of ultrasonic waves. Within the frequency range used to successfully pass through the skull, increasing frequencies enhances focality while reducing penetration depth. With the aid of neuronavigation, tFUS has penetration depths as far as 12 cm below the scalp [72] and focality within the range of 3 mm [73]. To best reach these parameters, in addition to optimized positioning of a stimulation transducer, fundamental frequency must be carefully tuned to successfully pass through the skull with minimal refraction, which can vary substantially due to individual morphological variability. For example, Lee et al. [74] found that, on average, only 18% of their intended tFUS energy reached their target, the primary visual cortex, with an average of 3.3 mm of focal deviation that could range as high as 16.1 mm. In terms of stimulation delivery, similar to rTMS, the majority of tFUS research uses pulsed stimulation protocols [75], which can help minimize health risks generated from excessive acoustic pressure, covered below. Within a single pulse of tFUS, the proportion of time in which acoustic energy is being delivered is known as a duty cycle. Pulse repetition frequency, then, is the pulse pattern structure (in Hz) within a stimulation train, and SD is the number of seconds it takes to deliver one complete train. Finally, intensity (W/cm^2^), also called energy flux density (EFD; mJ/mm^2^) or pressure (megapascals; MPa), is the total amount of ultrasound energy being absorbed within a unit of tissue space.

Because increases in these stimulation factors will tend to increase the amount of energy being introduced into brain tissue, the potential for harm increases as well. Although there are currently no established guidelines specific to ultrasound neuromodulation, tFUS research follows the safety parameters of the United States Food and Drug Administration (FDA) for diagnostic ultrasound. These guidelines serve to curb two primary health concerns involving ultrasound, which are excessive heating of tissue and potential cellular mechanical damage due to the cavitation phenomenon, which can result from pressure-based membrane tears and/or the formation and bursting of gas bubbles [76]. Accordingly, a thermal index is tracked by how much acoustic intensity is needed to raise tissue temperature by 1 °C while a mechanical index (MI) measures the potential for mechanical damage by dividing the peak negative pressure of an acoustic wave by the square root of its frequency [67,73,75]. These risks are minimal if proper safety protocols are followed, and FDA guidelines for diagnostic ultrasound limit acoustic intensity to 190 W/cm^2^ (or 7 MPa) and MIs to 1.9 [77]. Current human tFUS research utilizes low-intensity parameters within these ranges, typically between 0.5 and 100 W/cm^2^ [73] and MIs below 1.9 [67].

Negative side effects have been reported, though they are typically minimal and transient. Over the course of seven tFUS studies involving 64 total subjects, Legon et al. [78] had seven participants report mild to moderate side effects immediately following stimulation, which included neck pain, difficulty paying attention, muscle twitches, and/or anxiety. None were reported at follow-ups at one week and up to a month later. Other frequently reported symptoms across these studies included sleepiness, headaches, itchiness, tooth pain, and forgetfulness, though none were directly associated with stimulation. Another review on the safety of tFUS by Lee et al. [67] additionally noted no adverse effects in 13 human studies within standard safety parameters or in 13 animal studies using higher intensity stimulations of up to 25.8 W/cm^2^ and 4.62 MI.

## 3. Methods

This systematic review followed the Preferred Reporting Items for Systematic Reviews and Meta-Analyses (PRISMA) guidelines [79].

Relevant publications were identified in May 2025 through PubMed using combinations of the terms “Alzheimer’s disease”, “mild cognitive impairment”, or “dementia” with “clinical trial”, “randomized controlled trial”, “transcranial electrical stimulation”, “transcranial direct current stimulation”, “transcranial alternating current stimulation”, “transcranial magnetic stimulation”, and “transcranial ultrasound stimulation”. PubMed was selected as the primary database because it provides broad coverage of high-quality peer-reviewed biomedical and clinical neuroscience literature relevant to dementia and neuromodulation, and this search was supplemented by backward citation searching of relevant reviews and included articles to improve capture of additional studies. However, primarily restricting the search to PubMed may have reduced overall study coverage.

Titles and abstracts were screened first, followed by full-text review of potentially eligible papers. Studies were included if they examined TES, TMS, or tFUS in human participants with aMCI, MCI due to AD, prodromal AD, or AD and reported at least one cognitive, memory, or related neuropsychological outcome. Studies were excluded if they focused on non-AD dementias without clear AD-related diagnostic criteria, lacked a cognitive assessment component, were not original human studies, or had sample sizes too small for standard statistical analysis. Because the literature was highly heterogeneous, studies were grouped qualitatively by stimulation modality, target region, diagnostic stage, and where possible by study design (e.g., randomized sham-controlled, crossover, or open-label).

A formal risk-of-bias instrument was not applied because the review was designed as a qualitative, broad-scope synthesis of a highly heterogeneous literature spanning multiple stimulation modalities, study designs, target regions, and outcome measures rather than as a meta-analysis of directly comparable trials. Instead, interpretive emphasis was placed on whether studies used sham controls, randomization/blinding, clearly defined diagnostic groups, and follow-up assessments. This approach allowed major design features relevant to evidential strength to be considered, but it does not substitute for a formal structured bias assessment.

**Figure 1:**
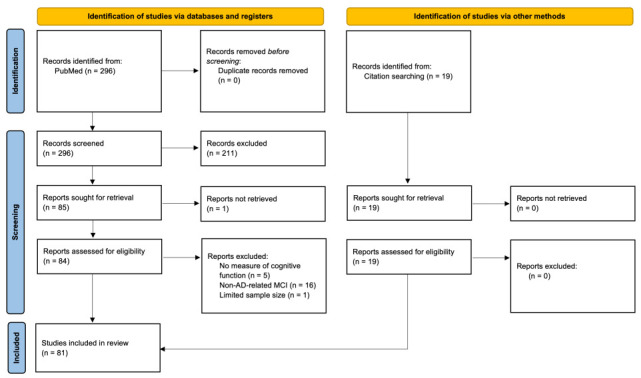
Flow chart of literature search and exclusion criteria.

A final total of 81 studies were included for review (Figure 1): 35 for TES, 38 for TMS, and 8 for tFUS. The main details and findings of each study are summarized in Table 1 (TES), Table 2 (TMS), and Table 3 (tFUS).

**Table 1:** Summary of TES studies.

Study	Population	Montage (Anode, Cathode)	Stim Parameters	Schedule	Improved Memory	Improved Assessment	Longevity	Online Effects
Lane et al. (2023) [80]	aMCI	Bilateral DLPFC, contralateral forehead	Anodal tDCS 2 mA, 20 min	10 daily sessions over 2 weeks		No significant differences between sham or verum		
Ladenbauer et al. (2017) [81]	aMCI	l-DLPFC and r-DLPFC, mastoids	Anodal, delta tDCS 0.75 Hz, 0.522 mA/cm^2^, 5 min/block, ISI 100 s, minimum 3 blocks	1 session	Visual recognition memory	Picture recognition task	Post-treatment	Closed-loop, NREM 2 delta band, additive (no sham group)
Murugaraja et al. (2017) [82]	aMCI	l-DLPFC, right supraorbital area	Anodal tDCS 2 mA, 20 min	5 consecutive daily sessions	Delayed and immediate recall	PMIT	Post-treatment, 1 month (no sham)	
Fileccia et al. (2019) [83]	aMCI	l-DLPFC, right deltoid	Anodal tDCS 2 mA, 20 min	20 daily sessions over 4 weeks	Global cognition, episodic memory, naming	BMDB, RAVLT immediate recall, figure naming	Post-treatment	
Alcalá-Lozano et al. (2025) [84]	aMCI	l-DLPFC, r-DLPFC	Anodal tDCS 2 mA, 30 min	15 daily sessions over 3 weeks		No significant differences compared to sham		CT (immediately after stimulation), non-additive
Šimko et al. (2024) [85]	aMCI	l-DLPFC, right middle frontal gyrus	Anodal tDCS 2 mA, 20 min	10 daily sessions, twice daily, over 2 weeks		No significant differences compared to sham		WMT, non-additive
Martin et al. (2019) [86]	aMCI	l-DLPFC, inferior frontal area	Anodal tDCS 2 mA, 30 min	15 sessions over 5–7 weeks	Verbal memory	CVLT (not significantly different from sham)	Post-treatment, 3 months	CT, non-additive
Antonenko et al. (2024) [87]	aMCI	l-DLPFC, contralateral supraorbital cortex	Anodal tDCS 1 mA, 20 min	9 sessions over 3 weeks	Working memory	NBT	Post-treatment	CT, trending
Manenti et al. (2020) [88]	aMCI	l-DLPFC, right supraorbital area	Anodal tDCS 1.5 mA, 15 min	1 session	Recall/recognition	Recall/recognition memory test	Real-time, 1 month	Task, additive
Rodella et al. (2022) [89]	aMCI and early AD	l-DLPFC, right deltoid	Anodal tDCS 2 mA, 30 min	12 daily sessions over 3 weeks	Working memory, attention	Working memory battery (VST, DST, CS), attention battery (AMT, TMT)	Post-treatment, 6 months (working memory)	CT, additive
Meléndez et al. (2023) [90]	AD	l-DLPFC, right frontal lobe	Anodal tDCS 2 mA, 20 min	5 consecutive daily sessions	Immediate and delayed recall	TAVEC	Post-treatment, 1 month	
Wang et al. (2024) [91]	Mild to moderate AD	l-DLPFC, right supraorbital area	Anodal tDCS 2 mA, 30 min	10 daily sessions over 2 weeks	Global cognition, working memory	MMSE, WCST	Post-treatment	
Khedr et al. (2014) [92]	Mild to moderate AD	l-DLPFC, contralateral supraorbital region	Anodal or cathodal tDCS 2 mA, 25 min	10 consecutive daily sessions	Global cognition, working memory	MMSE, DST (only cathodal)	Post-treatment, 2 months	
Rasmussen et al. (2021) [93]	AD	l-DLPFC	Anodal HD-tDCS 2 mA (anode), 0.5 mA (cathodes), 20 min, 15 min rest, 3 times	6 sessions over two days with 1–2 days of rest between	Delayed memory, global cognition	RBANS, MMSE	Post-treatment	
Suemoto et al. (2014) [94]	AD	l-DLPFC, above right orbit	Anodal tDCS 2 mA, 20 min	6 daily sessions every other day over 2 weeks		No significant differences compared to sham		
Cotelli et al. (2014) [95]	Mild to moderate AD	l-DLPFC, right deltoid	Anodal tDCS 2 mA, 25 min	10 daily sessions over 2 weeks	Associative memory (not significant from sham)	FNAT	Post-treatment, 12 weeks	CT, non-additive WMT, non-additive
Im et al. (2019) [96]	AD	l-DLPFC, r-DLPFC	Anodal tDCS 2 mA, 30 min	Daily sessions over 6 months	Global cognition, semantic memory	MMSE, BNT	Post-treatment	
Boggio et al. (2012) [97]	AD	Simultaneous bilateral temporal lobes, right deltoid	Anodal tDCS 2 mA, 30 min	5 consecutive daily sessions	Visual recognition memory	VRT	Post-treatment, 4 weeks	
Sprugnoli et al. (2021) [98]	Mild to moderate AD	Group 1—right temporal lobe (T8), 8 electrodes Groups 2 and 3—bilateral temporal lobes (P8, T8, P7, T7), 4 electrodes	Gamma tACS 40 Hz, 2 mA, 1 h	Groups 1–2–10 daily sessions over 2 weeks Group 3–20 daily sessions over 4 weeks		No significant changes in cognition/memory		Documentaries, non-additive
Khedr et al. (2019) [99]	Mild to moderate AD	Left then right temporal lobe, left deltoid arm	Anodal tDCS 2 mA, 20 min per side	10 daily sessions over 2 weeks	Global cognition, working memory, conceptual memory	3MS, MoCA, CDT	Post-treatment	
Zhou et al. (2022) [100]	AD	Bilateral temporal lobes, reference electrode not disclosed	Gamma tACS 40 Hz, 2 mA, 20 min	30 daily sessions over 6 weeks	Global cognition	MMSE, ADAS-Cog	Post-treatment, 12 weeks (MMSE)	
Lu et al. (2019) [101]	Mild neurocognitive disorder due to AD	Left lateral temporal cortex, contralateral upper limb	Anodal tDCS 2 mA, 20 min	12 daily sessions three times a week over 4 weeks	Delayed recall, working memory, logical memory	NBT DST	Post-treatment, 8 weeks (logical memory)	WMT, additive
Lu et al. (2025) [102]	Mild neurocognitive disorder due to AD	Left lateral temporal cortex, contralateral upper limb	Anodal tDCS 2 mA, 20 min	12 daily sessions three times a week over 4 weeks	Memory (poor sleepers)	ADAS-Cog	Post-treatment, 8 weeks	WMT, additive
Bystad et al. (2016) [103]	AD	Left temporal lobe, right frontal lobe	Anodal tDCS 2 mA, 30 min	6 daily sessions over 10 days	Delayed recall (trending)	CVLT-II	Post-treatment	
Gangemi et al. (2021) [104]	AD	Left frontotemporal lobe, right frontal lobe	Anodal tDCS 2 mA, 20 min	Study 1: 10 daily sessions Study 2: 10 consecutive daily sessions per month over 8 months	Global cognition	MMSE (stable, not improved)	Post-treatment	
Benussi et al. (2021) [105]	aMCI-AD	Precuneus, right deltoid	Gamma tACS 40 Hz, 1.5 mA, 60 min	1 session	Episodic memory, associative memory	RAVLT, FNAT	Post-treatment	FNAT, additive
Benussi et al. (2022) [106]	AD	Precuneus, right deltoid	Gamma tACS 40 Hz, 1.5 mA, 60 min	1 session	Episodic memory, associative memory	RAVLT, FNAT	Post-treatment	FNAT, additive
Hu et al. (2022) [107]	AD	Bilateral angular gyrus, contralateral frontal area	rTMS + anodal tDCS 90% rMT 40 Hz + 2 mA, 15 min per side	12 daily sessions every other day over 4 weeks	Global cognition	MMSE, ADAS-Cog	Post-treatment, 8 weeks	
LoBue et al. (2025) [108]	AD	Medial prefrontal cortex	Anodal HD-tDCS 1 or 2 mA, 20 min	10 daily sessions over 2 weeks	Episodic memory, phonemic fluency	RAVLT, DKEFS phonemic fluency (not significantly different from sham)	Post-treatment, 8 weeks (1 mA)	
Tang et al. (2024) [109]	Mild AD	Hippocampus (conductive pad over Fpz, Fp1, Fp2, conductive pads over each mastoid)	Gamma tACS 40 Hz, 15 mA, 1 h	30 daily sessions twice a day (4 h interval) over 15 days	Global cognition	MMSE, MoCA	Post-treatment	
Andrade et al. (2022) [110]	AD	NeuroAD, contralateral supraorbital area	Anodal tDCS 2 mA, 10 min per brain area	24 daily sessions, 3 times a week over 2 months	Global cognition	ADAS-Cog	Post-treatment	CT, additive
de Sousa et al. (2020) [111]	aMCI	Right temporoparietal cortex, left supraorbital area	Anodal tDCS 1 mA, 20 min	3 consecutive daily sessions in each condition	Object-location memory	Object-location memory training and recall	Post-treatment	Object-location memory training, additive (non-sham-controlled)
Jones et al. (2023) [112]	aMCI	Prefrontal cortex (Humm patch)	Theta tACS 6 Hz, 1.5 mA, 16 min	5 consecutive daily sessions followed by 3 weekly maintenance sessions	Attention, inhibitory control	ACE-X reaction time, ST		CT, additive
Philippen et al. (2024) [113]	aMCI and AD	Right temporoparietal junction (2 anode electrodes), cathodes on (CP4, T8, P10), reference electrodes on right mastoid	Anodal tDCS 2 mA, 20 min	1 session	Spatial memory	Virtual water maze	Post-treatment	Task, additive
Meinzer et al. (2015) [114]	aMCI	l-vIFG, right supraorbital region	Anodal tDCS 1 mA, 20 min	1 session	Semantic memory	Semantic word generation task	Real-time	Additive

Note. AD = Alzheimer’s disease, aMCI = amnestic mild cognitive impairment, tDCS = transcranial direct current stimulation, tACS = transcranial alternating current stimulation, DLPFC = dorsolateral prefrontal cortex, PMIT = Picture Memory Impairment Test, BMDB = Brief Mental Deterioration Battery, RAVLT = Rey Auditory Verbal Learning Test, CVLT = California Verbal Learning Task, NBT = N-Back Task, VST = Verbal Span Test, DST = Digit Span Test, CS = Corsi Span, AMT = Attentive Matrices Test, TMT = Trail-Making Task, TAVEC = Spanish version of the California Verbal Learning Test, MMSE = Mini-Mental State Examination, WCST = Wisconsin Card Sorting Test, RBANS = Repeatable Battery for the Assessment of Neuropsychological Status, FNAT = Face–Name Association Test, BNT = Boston Naming Test, VRT = Visual Recognition Task, 3MS = Modified Mini-Mental State Examination, MoCA = Montreal Cognitive Assessment, CDT = Clock-Drawing Task, ADAS-Cog = Alzheimer’s Disease Assessment Scale—Cognitive Subscale, DKEFS = Delis–Kaplan Executive Function System, ACE-X = Adaptive Cognitive Evaluation–Explorer, ST = Stroop Task.

**Table 2:** Summary of TMS studies.

Study	Population	Montage	Stim Parameters	Schedule	Improved Memory	Improved Assessment	Longevity	Online Effects
Drumond Marra et al. (2015) [115]	aMCI	l-DLPFC	110% rMT 10 Hz, 5 s, 25 ISI, 2000 pulses	10 consecutive daily sessions	Memory	RBMT	Post-treatment, 30 days	
Bagattini et al. (2020) [116]	aMCI and mild to moderate AD	l-DLPFC	100% rMT 20 Hz, 2 s, 28 s ISI, 2000 pulses	20 sessions over 4 weeks	Associative memory, visuospatial reasoning	RCPM	Post-treatment, 3 months	CT, additive
Aghamoosa et al. (2024) [117]	aMCI	l-DLPFC	120% rMT 50 Hz, 2 s, 8 s ISI, 600 pulses	8 stim sessions daily over 3 optionally non-consecutive days	Global cognition	NIHTB-CB	Post-treatment	
Wu et al. (2015) [118]	AD	l-DLPFC	80% rMT 20 Hz, 1200 pulses	20 daily sessions over 4 weeks	Global cognition	ADAS-Cog	Post-treatment	
Li et al. (2021) [119]	Mild to moderate AD	l-DLPFC	100% rMT 20 Hz, 1 s, 10 s ISI, 2000 pulses	30 daily sessions over 6 weeks	Global cognition	ADAS-Cog, MMSE	Post-treatment, 3 months	
Tao et al. (2022) [120]	AD	l-DLPFC	100% rMT 20 Hz, 2 s, 25 ISI, 1760 pulses	30 daily sessions over 6 weeks	Global cognition	MMSE, MoCA, ADAS-Cog	Post-treatment	
Cotelli et al. (2011) [121]	Moderate AD	l-DLPFC	100% rMT 20 Hz, 2 s, 28 ISI, 2000 pulses	10 daily sessions over 2 weeks	Language	Sentence comprehension in BAAD	Post-treatment, 8 weeks	
Padala et al. (2020) [122]	AD	l-DLPFC	120% rMT 10 Hz, 4 s, 26 s ISI, 3000 pulses	20 daily sessions over 4 weeks	Global cognition	3MS	Post-treatment, 8 weeks	
Zhang et al. (2019) [123]	Mild to moderate AD	l-DLPFC and left lateral temporal lobe	100% rMT 10 Hz, 5 s, 25 ISI, 1000 pulses per brain region	20 daily sessions over 4 weeks	Global cognition	ADAS-Cog	Post-treatment, 4 weeks	CT, additive
Zhang et al. (2023) [124]	Moderate to severe AD	l-DLPFC	100% rMT 10 Hz, 4 s, 16 ISI, 2400 pulses	3 sets of 20 consecutive daily sessions, separated by 10 days	Global cognition (severe impairment)	SIB	Post-treatment	
Lin et al. (2024) [125]	AD	l-DLPFC	80% rMT 50 Hz, 2 s, 8 s ISI, 1800 pulses	2 sessions per day, 14 consecutive days	Verbal memory	AVLT	Post-treatment	
Wu et al. (2022) [126]	AD	l-DLPFC	70% rMT 50 Hz, 600 pulses, 3 sessions per day	14 consecutive daily sessions	Associative memory, global memory, attention, language and verbal memory, executive function, global cognition	Face-cued word association test, MoCA, MMSE, LMT, AVLT, DST, SDMT, SCWT, CDT, HVOT, JOLT, BNT, VFT	Post-treatment, 8 weeks	
Cotelli et al. (2008) [127]	Mild, moderate, and severe AD	l-DLPFC, r-DLPFC	90% rMT 20 Hz, 500 ms per stimulus	1 session	Semantic	Action–object picture naming task	Real-time	Task, additive
Cui et al. (2019) [128]	aMCI	r-DLPFC	90% rMT 10 Hz, 5 s, 25 s ISI, 1500 pulses	10 daily sessions over 2 weeks	Global cognition	AVLT	Post-treatment, 8-week follow-up	
Ahmed et al. (2012) [129]	AD	Bilateral DLPFC (right, then left)	90% rMT 20 Hz, 5 s, 25 s ISI, 2000 pulses 100% rMT 1 Hz, two trains, 30 s ISI, 2000 pulses	5 consecutive daily sessions	Global cognition	MMSE	Post-treatment, 3 months (greater in 20 Hz)	
Moussavi et al. (2024) [130]	Mild to moderate AD	Bilateral DLPFC (l, then r)	90–100% rMT 20 Hz, 1.5 s, 10 ISI, 1500 pulses	10 daily sessions over 2 weeks or 20 daily sessions over 4 weeks	Global cognition (not significantly different from sham)	ADAS-Cog	Post-treatment, 6 months	
Rutherford et al. (2015) [131]	Early and advanced AD	Bilateral DLPFC	90–100% rMT 20 Hz, 2 s, 5 s ISI, 2000 pulses per hemisphere	10 daily sessions over 2 weeks 3 additional daily sessions over 2 weeks 10 additional verum daily sessions over 2 weeks every 2–7 months for 19 months total	Global cognition	MoCA	Post-treatment (after 2 and 3 weeks)	Identified objects between pulses, but no control to compare to
Zhou et al. (2022) [132]	AD	Bilateral DLPFC	120% rMT 10 Hz, 1500 pulses (l-DLPFC) 1 Hz, 1500 pulses (r-DLPFC)	20 daily sessions over 4 weeks	Global cognition	ADAS-Cog	Post-treatment, 8 weeks	
Bentwich et al. (2011) [133]	Early or moderate AD	NeuroAD	90% rMT Broca’s area, l/r-DLPFC 110% rMT Wernicke’s area, l/r-pSAC 10 Hz, 2 s, 400 pulses per brain area	30 daily sessions over 6 weeks 24 biweekly sessions for 3 months after	Global cognition	ADAS-Cog, MMSE	Post-treatment	CT, not sham-controlled
Nguyen et al. (2017) [134]	AD	NeuroAD and l/r-PFC	100% rMT 10 Hz, 2 s, 400 pulses per brain area Additional 10 Hz, 2 s, 100 pulses for l/r-DLPFC	25 daily sessions over 5 weeks	Global cognition	ADAS-Cog	Post-treatment, 6 months	CT, not sham-controlled
Rabey et al. (2013) [135]	Mild to moderate AD	NeuroAD	90% rMT Broca’s area, l/r-DLPFC 110% rMT Wernicke’s area, l/r-pSAC 2 brain areas 10 Hz, 2 s, 400 pulses 1 brain area 10 Hz, 2 s, 500 pulses	30 daily sessions over 6 weeks, biweekly maintenance for 3 months, 54 sessions total	Global cognition	ADAS-Cog	Post-treatment (intensive and maintenance)	CT, additive
Lee et al. (2016) [136]	Mild and moderate AD	NeuroAD	90% rMT Broca’s area, l/r-DLPFC 110% rMT Wernicke’s area, l/r-pSAC 10 Hz, 2 s, 400 pulses per brain area	30 daily sessions over 6 weeks	Global cognition	ADAS-Cog	Post-treatment, 6 weeks	CT, additive
Sabbagh et al. (2020) [137]	Mild to moderate AD	NeuroAD	110% rMT 10 Hz, 1300 total pulses across 3 brain areas	30 daily sessions across 6 weeks	Global cognition	ADAS-Cog	Post-treatment, 6 weeks	CT, non-additive until 6 weeks later
Brem et al. (2020) [138]	Mild to moderate AD	NeuroAD	120% rMT 10 Hz, 2 s	30 daily sessions over 6 weeks	Global cognition	ADAS-Cog	Post-treatment, 4–6 weeks	CT, additive (follow-up)
Vecchio et al. (2022) [139]	Mild to moderate AD	NeuroAD	90% rMT (frontal cortex) 110% rMT (other regions) 10 Hz, 2 s, 1200–1400 pulses	30 daily sessions over 6 weeks	Global cognition	ADAS-Cog	Post-treatment, 40 weeks (verum)	CT, additive (after 40 weeks)
Alcalá-Lozano et al. ( 2018) [140]	AD	l-DLPFC or NeuroAD-like therapy (no CT)	100% rMT 5 Hz, 10 s, 60 s ISI, 1500 pulses (500/area)	15 daily sessions over 3 weeks	Global cognition	ADAS-Cog, MMSE	Post-treatment, 4 weeks	
Koch et al. (2018) [141]	Prodromal AD	Precuneus	100% rMT 20 Hz, 2 s, 28 ISI, 1600 pulses	10 daily sessions over 2 weeks	Episodic memory	RAVLT delayed recall	Post-treatment	
Jung et al. (2024) [142]	aMCI Mild AD dementia	Precuneus	100% rMT 20 Hz, 2 s, 1600 pulses	5 daily sessions per week over 4 weeks	Global cognition	ADAS-Cog	Post-treatment, 4 weeks	
Koch et al. (2022) [143]	Mild to moderate AD	Precuneus	100% rMT 20 Hz, 2 s, 28 ISI, 1600 pulses	10 daily sessions over 2 weeks, followed by 22 weekly sessions	Global cognition	ADAS-Cog, MMSE	Week 12, Week 24	
Koch et al. (2025) [144]	Mild to moderate AD	Precuneus	100% rMT 20 Hz, 2 s, 28 ISI, 1600 pulses	10 daily sessions over 2 weeks, followed by 50 weekly sessions	Global cognition	ADAS-Cog, MMSE	Week 12, Week 24, Week 36, Week 52	
Chen et al. (2023) [145]	aMCI and AD	Left angular gyrus	100% rMT 20 Hz, 2 s, 28 ISI, 1600 pulses	20 daily sessions over 4 weeks	Global cognition, global memory	MoCA-BJ, memory composite z-score	Post-treatment	
Liu et al. (2022) [146]	AD	Left and right angular gyrus	40% rMT 40 Hz, 2 s, 58 s ISI, 2400 pulses	12 daily sessions every other weekday over 4 weeks	Global cognition	ADAS-Cog, MMSE, MoCA	Post-treatment, 8 weeks	
Jia et al. (2021) [147]	AD	Left lateral parietal cortex	100–110% rMT 10 Hz, 2 s, 28 ISI, 800 pulses	10 daily sessions over 2 weeks	Verbal and episodic memory, global cognition	PVLT MMSE	Post-treatment	
Wei et al. (2022) [148]	Mild to moderate AD	Custom site in lateral parietal lobule with highest functional connectivity to hippocampus	100–110% rMT 10 Hz, 2 s, 28 s ISI, total pulses not disclosed	10 daily sessions over 2 weeks	Global cognition Verbal and episodic memory	MMSE, PVLT	Post-treatment	
Zhao et al. (2016) [149]	Mild and moderate AD	Parietal and posterior temporal lobe (P3/P4, T5/T6)	rMT not disclosed 20 Hz, 10 s, 20 s ISI, 20–40 s per brain area (3 per session)	30 daily sessions over 6 weeks	Global cognition, verbal memory	ADAS-Cog, MMSE, MoCA, AVLT	Post-treatment, 6 weeks	CT, additive
Hoy et al. (2023) [150]	AD	l-DLPFC, r-DLPFC, l-PPC, r-PPC	100% rMT 50 Hz, 2 s, 10 ISI, 600 pulses	21 sessions over 6 weeks	Episodic memory	ISL delayed recall	Post-treatment	
Eliasova et al. (2014) [151]	aMCI and early AD	r-IFG, r-STG	90% rMT 10 Hz, 4.9 s, 25 s ISI, 2250 pulses	3 sessions with a day between sessions	Working memory	TMT-A and B	Post-treatment	
Yao et al. (2022) [152]	AD	Bilateral cerebellum	90% rMT 5 Hz, 2000 pulses	20 daily sessions over 4 weeks	Global cognition, verbal memory, episodic memory, executive ability, verbal ability, visuospatial function	MMSE, MoCA, ADAS-Cog, RAVLT, CDT, BNT, VFT, TMT-A/B, DST, SDMT	Post-treatment, 8 weeks	

Note. AD = Alzheimer’s disease, aMCI = amnestic mild cognitive impairment, rMT = resting motor threshold, ISI = interstimulus interval, DLPFC = dorsolateral prefrontal cortex, PPC = posterior parietal cortex, IFG = inferior frontal gyrus, RBMT = Rivermead Behavioral Memory Test, RCPM = Raven Colored Progressive Matrices, NIHTB-CB = NIH Toolbox Cognition Battery, ADAS-Cog = Alzheimer’s Disease Assessment Scale—Cognitive Subscale, MMSE = Mini-Mental State Examination, BAAD = Battery for Analysis of Aphasic Deficits, 3MS = Modified Mini-Mental State Examination, SIB = Severe Impairment Battery, AVLT = Auditory Verbal Learning Task, MoCA = Montreal Cognitive Assessment, LMT = Logical Memory Test, DST = Digit Span Test, SDMT = Symbol Digit Modalities Test, SCWT = Stroop Color and Word Test, CDT = Clock-Drawing Task, HVOT = Hooper Visual Organization Test, JOLT = Judgment of Line Orientation Test, BNT = Boston Naming Test, VFT = Verbal Fluency Test, RAVLT = Rey Auditory Verbal Learning Test, MoCA-BJ = Montreal Cognitive Assessment (Beijing), PVLT = Philadelphia Verbal Learning Test, ISL = International Shopping List, TMT = Trail-Making Test.

**Table 3:** Summary of tFUS studies.

Study	Population	Montage	Stim Parameters	Schedule	Improved Memory	Improved Assessment	Longevity
Beisteiner et al. (2019) [153]	AD	Site 1: Bilateral frontal cortex, bilateral lateral parietal cortex, extended precuneus cortex Site 2: Evenly across scalp	PRF = 5 Hz EFD = 0.2 mJ/mm^−2^ 6000 pulses Site 1: Frontal cortex—800 pulses per hemisphere, twice Lateral parietal cortex—400 pulses per hemisphere, twice Precuneus—600 pulses twice	3 sessions per week for 2–4 weeks	Global cognition	CERAD	Post-treatment, 3 months
Popescu et al. (2021) [154]	AD	Bilateral frontal cortex, bilateral parietal cortex, extended precuneus cortex	PRF = 5 Hz EFD = 0.2 mJ/mm^−2^ 6000 pulses Site 1: Frontal cortex—800 pulses per hemisphere, twice Lateral parietal cortex—400 pulses per hemisphere, twice Precuneus—600 pulses twice	3 sessions per week for 2–4 weeks	Global cognition	CERAD	Post-treatment, 3 months
Dörl et al. (2022) [155]	AD	Bilateral frontal cortex, bilateral parietal cortex, extended precuneus cortex	PRF = 5 Hz EFD = 0.2 mJ/mm^−2^ 6000 pulses Site 1: Frontal cortex—800 pulses per hemisphere, twice Lateral parietal cortex—400 pulses per hemisphere, twice Precuneus—600 pulses twice	3 sessions per week for 2–4 weeks	Global cognition (worsened in non-stimulated areas)	CERAD (worsened in non-stimulated areas)	Post-treatment (trending), 3 months
Matt et al. (2025) [156]	AD, MCI, dementia in AD or MCI	Bilateral frontal cortex, bilateral parietal cortex, extended precuneus cortex	PRF = 5 Hz DC = 0.0015% EFD = 0.30 mJ/mm^2^ Intensity = 24 mW/cm^2^ 20 min 6000 pulses	6 daily sessions over 2 weeks	Global cognition	CERAD (only younger than 70)	Post-treatment, 3 months
Cont et al. (2022) [157]	Mild to severe AD	Bilateral frontal cortex, bilateral parietal cortex, bilateral temporal cortex, extended precuneus cortex	PRF = 4 Hz EFD = 0.20 mJ/mm^2^ 6000 pulses every two days for six sessions over 2 weeks or 3000 pulses every day over 12 sessions	See left	Global cognition	ADAS-Cog	Post-treatment
Shinzato et al. (2024) [158]	Mild to moderate AD	Frontotemporal, parietal, occipital regions	PRF = 4 Hz EFD = 0.25 mJ/mm^2^ 6000 pulses	Twice a week for 5 consecutive weeks	Global cognition	ADAS-Cog	Post-treatment (non-significant), 90 days (trending)
Shimokawa et al. (2022) [159]	Early-stage AD (aMCI or mild AD)	Whole brain	PRF = 781 Hz FF = 0.5 MHz DC = 5% Intensity = 1.3 MPa 20 min, 3 times per session with 5 min between stims	Every other day for three days a week every 3 months over 18 months		No significant differences compared to sham	
Jeong et al. (2022) [160]	Moderate to severe AD	Right hippocampus	PRF = 2 Hz FF = 250 kHz DC = 4% SD = 300 ms Intensity = 3.0 W/cm^2^ 180 s	1 session	Immediate recall, recognition memory	VLT	Post-treatment

Note. AD = Alzheimer’s disease, aMCI = amnestic mild cognitive impairment, PRF = pulse repetition frequency, EFD = energy flux density, FF = fundamental frequency, DC = duty cycle, SD = sonication duration, CERAD = Consortium to Establish a Registry for Alzheimer’s Disease, ADAS-Cog = Alzheimer’s Disease Assessment Scale—Cognitive Subscale, VLT = Verbal Learning Test.

## 4. Results of TES Studies

### 4.1. Dorsolateral Prefrontal Cortex (DLPFC)

The most commonly stimulated brain region across the reviewed TES studies was the DLPFC. A total of 15 studies exclusively stimulated the left hemisphere (l-DLPFC) while 2 others applied stimulation bilaterally. Save for one, in which cathodal stimulation was also used, all studies used anodal tDCS stimulation targeted to the DLPFC, with intensities of 1–2 mA (though most used 2 mA) for 15–30 min. Unless specified, it should be assumed tDCS is anodal for this section, as cathodal DLPFC stimulation was only used in one of the following studies.

These studies showed mixed results. Lane et al. [80] used 10 tDCS sessions applied to the bilateral DLPFC over 2 weeks in early-stage AD patients. There were no significant differences in cognitive or memory assessments across stimulation or drug conditions (daily sodium benzoate). Ladenbauer et al. [81] also applied anodal tDCS over the bilateral DLPFC, and in addition included oscillating 0.75 Hz stimulation during the slow-wave phase of sleep during a 90 min daytime nap, which enhanced slow-wave activity in the delta band. Verum stimulation was associated with improved visual recognition memory and increased power in frequencies associated with slow oscillation (0.5–1 Hz) and thalamocortical sleep spindles (12–15 Hz). Murugaraja et al. [82] also found immediate and delayed recall were improved following five consecutive daily sessions of 2 mA tDCS, which were further improved or stable at a follow-up one month later. Over a longer course of 20 daily sessions over 4 weeks, Fileccia et al. [83] found significant improvements to global cognition, immediate episodic verbal memory, and figure naming upon completion of the treatment regimen.

Online tDCS generally did not provide significant additive effects to memory or cognition when cognitive training (CT) tasks or working memory training (WMT) were also administered in aMCI patients. Immediately following stimulation in 9 of 15 daily sessions over 3 weeks, subjects in Alcalá-Lozano et al. [84] received cognitive stimulation in group settings. No additive effects were found on cognitive tests or biological markers, however. In another study by Šimko et al. [85] using WMT online with stimulation, verum subjects performed no better than sham subjects after 10 daily sessions (twice daily) over 5 days or at follow-up a month later. Although there were no significant differences between verum and sham groups in Martin et al. [86], the authors did find that, from baseline, verum subjects improved significantly in the California Verbal Learning Task after 15 daily sessions over 5 weeks, while sham subjects did not. Both groups made large improvements 3 months post-treatment, as well, though neither was greater than the other. Despite only trending additive effects in an N-Back Task (NBT), Antonenko et al. [87] reported functional connectivity significantly increased in the frontoparietal network of aMCI patients following nine sessions of 20 min, 1 mA tDCS.

Manenti et al. [88], however, did report an additive effect of 1.5 mA verum tDCS to CT, which transpired over only a single session. In their cohort of aMCI subjects, they additionally noted that, at a 1-month follow-up, the verum group’s recall and recognition abilities were comparable to that of the sham average immediately after they had received CT. Additionally, 12 daily sessions over 3 weeks improved the working memory and attention of subjects in Rodella et al. [89], which remained further improved at follow-up 6 months later. MMSE scores also remained stable at follow-up whereas sham subjects’ scores significantly worsened.

Regarding TES in AD patients, Meléndez et al. [90] found immediate and delayed recall were improved in the Test de Aprendizaje Verbal España-Complutense (a Spanish-language verbal learning test) following five consecutive daily sessions of 2 mA tDCS and were further improved or stable at a follow-up one month later. Mini-Mental State Examination (MMSE) scores were additionally improved in verum compared to sham, although not until the follow-up session. In mild to moderate AD patients, Wang et al. [91] reported improved abstract reasoning, as well as cognitive flexibility and function, following 10 weekday, 2 mA tDCS sessions over 2 weeks when subjects received verum stimulation compared to when they received sham stimulation. In Khedr et al. [92], both anodal and cathodal 2 mA tDCS significantly improved MMSE scores following 10 consecutive daily sessions in mild to moderate AD adults. Although both were improved compared to sham, the results of the two verum stimulation types were not significantly different from each other. Digit span performance scores on the Wechsler Adult Intelligence Scale (WAIS-III) were also significantly improved, but only in the cathodal group compared to sham. Regardless, across all assessments, these effects remained stable at follow-ups up to 2 months later. In another study by Rasmussen et al. [93], 2 mA HD-tDCS significantly improved MMSE scores and delayed memory abilities after six sessions given over the course of 2 days in the verum group compared to the sham group. Suemoto et al. [94] did not find significant improvements in memory or cognitive domains after six daily sessions of 2 mA tDCS over 2 weeks. In the only study using an online component with AD patients, Cotelli et al. [95] also did not find significant additive effects of one 2 mA tDCS session on generalized cognition or memory after a CT was administered during stimulation. However, both CT groups performed better than a control tDCS + motor training group.

Notably, one study applied 6 months of daily 2 mA tDCS at home. Compared to sham, AD patients who received verum stimulation in Im et al. [96] had significant improvements in MMSE and Boston Naming Task (BNT) scores. Additionally, executive function was stabilized in those receiving verum stimulation while a marked decrease was observed in the sham group.

### 4.2. Temporal Lobes/Cortex

A total of eight studies applied TES to the temporal lobe. Four used bilateral stimulation while the other four only left temporal lobe stimulation, although stimulation of the right temporal lobe was also used in a separate cohort in one of the bilateral studies. One of the studies used anodal 2 mA tDCS, while the others used 2 mA gamma (40 Hz) tACS. All studies involved only AD patients with stimulation times ranging from 20 min to a full hour.

Several studies reported beneficial effects of bilateral temporal lobe stimulation. Boggio et al. [97] used a crossover design featuring five consecutive daily sessions of bilateral tDCS to the temporal lobes. Significant improvements in visual recognition memory after verum tDCS were made, which persisted 4 weeks later. Comparatively, when subjects received sham stimulation, there was a notable decline. In another simultaneous bilateral stimulation study, Sprugnoli et al. [98] featured an online component of documentaries alongside an hour of 40 Hz tACS, though no significant changes in cognition or memory ability were reported after 2 or 4 weeks of treatment. Compared to sham stimulation, verum stimulation significantly increased blood flow to the right medial temporal pole, fusiform gyrus, and entorhinal cortex, as well as gamma band power within the right temporal lobe. Meanwhile, Khedr et al. [99] assessed the differences in Aβ plasma levels before and after treatment. Compared to sham, those who received verum anodal tDCS to each temporal lobe had significant improvement in Aβ levels and the Modified MMSE (3MS) and Montreal Cognitive Assessment (MoCA) assessments. Clock-Drawing Task (CDT) scores were also improved, and serum Aβ levels were also significantly correlated with all of the above improvements. Similar effects were found in Zhou et al. [100] as MMSE and ADAS-Cog scores were improved from baseline following 30 daily sessions spread over 6 weeks in the verum group, while Aβ levels significantly decreased in blood samples. MMSE scores continued to improve 12 weeks later.

Only one study used online anodal tDCS over the left temporal lobe. Lu et al. [101] paired WMT with either sham or verum tDCS. The study also included a third group who received verum tDCS and a control for WMT that tested attention. Treatment was spread over 12 sessions over 4 weeks. All three groups improved in the Alzheimer’s Disease Assessment Scale—Cognitive Subscale (ADAS-Cog) and NBT performance, and these effects carried over at 4- and 8-week follow-ups. Those who received WMT and verum tDCS, however, made greater post-treatment improvements in delayed recall and working memory capacity aspects of the NBT, as well as improvements in a forward Digit Span Task assessing logical memory that held 8 weeks later. A follow-up analysis of the same study by Lu et al. [102] later noted that poor sleepers benefitted significantly more from verum tDCS than good sleepers in their total ADAS-Cog scores, with poor sleepers in the WMT group outperforming good sleepers up to 8 weeks later. Though poor sleepers in the control WMT group only outperformed good sleepers for 4 weeks after, they also made significant improvements in their total Pittsburgh Sleep Quality Index (PSQI) scores that persisted 8 weeks later.

Regarding offline stimulation of the left temporal lobe, no significant effects on memory or cognition were observed in AD patients following anodal tDCS of the left temporal lobe in Bystad et al. [103]. Though MMSE and Milan Overall Dementia Assessment (MODA) scores were also not improved in Gangemi et al. [104], they remained stable in the verum group whereas they declined significantly in the sham group. This was true in the short-term first part of the study, in which 10 daily sessions were applied over the left frontotemporal lobe, as well in a longer second portion where 10 consecutive daily sessions were given every month for eight months. Additionally, these effects featured corresponding enhanced alpha, beta, and theta band power in both studies under the verum group, while they worsened in sham.

### 4.3. Other Brain Regions

Ten other TES studies on aMCI and AD patients were gathered targeting other brain regions. Stimulation intensities ranged from 1 to 2 mA in all but one of the studies, in which 15 mA current was delivered. Stimulation times lasted between 16 min to a full hour.

Three studies targeted areas in the parietal cortex in AD patients. Benussi et al. [105,106] stimulated the precuneus with 1.5 mA of 40 Hz tACS with online CT components for associative memory. A mix of MCI and AD were in the former [105] while only AD patients were in the latter [106]. After one session, significant improvements were made in the Rey Auditory Verbal Learning Test (RAVLT) and the Face–Name Association Test (FNAT) when verum stimulation was applied for a full hour. Improved cholinergic transmission was observed as well, and Benussi et al. [106] additionally found significant positive correlations between the amount of current reaching the precuneus and the degree of improvements made in memory. Meanwhile, in a unique study combining multiple forms of NIBS, Hu et al. [107] targeted the bilateral angular gyrus. The combination of tDCS with 40 Hz rTMS led to significant improvements in MMSE scores, with a larger combined effect compared to when both NIBS techniques were applied individually. These effects remained stable or improved at a follow-up session 8 weeks after the final stimulation session (12 daily sessions over 4 weeks). ADAS-Cog scores were also improved, though not more in comparison to isolated tDCS or rTMS. PSQI scores in the rTMS + tDCS group were significantly more improved in comparison to single rTMS and trending for single tDCS at both time points as well.

Two other AD studies targeted more frontal regions of the brain. To compare different stimulation intensities, LoBue et al. [108] applied 1 or 2 mA of anodal (or sham) HD-tDCS over the medial prefrontal cortex. No significant differences were found in memory or cognition between the two intensities and sham after 10 daily sessions were given over 2 weeks, nor at an 8-week follow-up. However, large effect sizes in RAVLT and phonemic fluency improvement were observed in the verum groups post-treatment, which were maintained up to 8 weeks later in the 1 mA but not the 2 mA group. In mild AD patients receiving 40 Hz tACS over the frontopolar cortex, compared to sham, the verum subjects in Tang et al. [109] had significant improvements in MMSE and MoCA scores after 30 twice-daily sessions over 15 days. These cognitive test gains were correlated with enhanced theta–gamma activity thought to be emitted by the hippocampus. However, the effects did not persist at a 3-month follow-up. Notably, this study used a high 15 mA intensity. Significant negative adverse events were not reported by the authors.

Finally, Andrade et al. [110] used a split A/B session schedule in which Broca’s area, Wernicke’s area, and the r-DLPFC were stimulated on “A” days while the l-DLPFC and the left and right somatosensory association cortices (SACs) were stimulated on “B” days. Additionally, all subjects were given online cognitive tasks associated with the region being stimulated in the moment. Over the course of 24 daily sessions over 2 months, each region received 10 min of 2 mA anodal tDCS per session, and significant improvements were made in ADAS-Cog scores in both verum and sham groups. There was a greater effect in the verum group, however, which was also reflected in increased delta, theta, alpha, and beta band power, particularly in those with milder AD.

Regarding aMCI populations, when compared to healthy adults, patients in de Sousa et al. [111] made comparable gains in a CT task when they received 1 mA verum tDCS at the temporoparietal cortex, though the effects of CT or the facilitation of TES did not persist after one month. In verum patients compared to sham, Jones et al. [112] found no significant added effect of theta (6 Hz) tACS over the prefrontal cortex to CT in aMCI patients in cognitive or memory domains. The verum subjects did, however, make greater improvements in inhibitory control and ability to sustain attention from baseline after 5 days of treatment, which were maintained a month later following weekly maintenance sessions. In a mixed AD/MCI subject pool, one 20 min session of 2 mA tDCS over the right temporoparietal junction significantly improved recall abilities in a virtual navigation task in Philippen et al. [113]. Additionally, it was found that cognitively impaired subjects who received verum stimulation had immediate recall capabilities similar to healthy controls despite notable atrophy in the hippocampus. In Meinzer et al. [114], a single online session of verum tDCS over the left inferior frontal gyrus brought aMCI subjects’ performance on a semantic word generation task up to levels comparable to healthy controls, while sham aMCI subjects remained significantly lower than either of the two other groups. Neuroimaging also found that task-based regional hyperactivity was reduced in subjects who received verum stimulation while resting-state functional connectivity was modified in multiple regions of interest, showing patterns in both instances more aligned with healthy controls.

### 4.4. Discussion of TES Studies

In addition to improvements in general cognition, domain-specific improvements in working memory, episodic memory, associative memory, visual recognition memory, delayed and immediate recall, semantic memory, attention and inhibitory control, conceptual memory, and logical memory both online and later offline were made across many but not all TES studies in aMCI and AD patients reviewed here. The DLPFC was the most stimulated region of interest, commonly cited as a target due to it being a network hub for real-time cognitive processes involving working memory and executive function, which could facilitate diffusion of neuromodulation. Two other hub systems, the precuneus and angular gyrus, also provided promising results in enhancing memory, with the particular combination of 40 Hz rTMS and tDCS leading to long-lasting effects on two tests of global cognition at 8 weeks post-treatment in the latter [107]. While online components such as cognitive training generally were mixed in providing additive effects to TES in terms of behavioral results, greater neuronal and network-level improvements in the regions of interest were reported in all tDCS and tACS online studies that employed neuroimaging, suggesting that stimulation is nonetheless providing beneficial effects to the underlying brain circuitry at work, even after just one session.

On that note, several studies used 40 Hz tACS in temporal and parietal regions of interest, finding increase blood flow to temporal areas, gamma band activity in the hippocampus, cholinergic transmission in the precuneus, and clearance of Aβ as assessed by blood analysis [98,100,106,109]. Processes of cognition, memory, and attention have been associated with gamma band activity in the cortico-hippocampal network [161], and, in mice models of AD, enhancement of gamma activity has also been associated with morphological microglial changes more conducive to phagocytosis of Aβ [162]. Restoring some degree of function to gamma networks appears possible through TES, and due to the development of neurofibrillary changes and amyloid accumulation in the temporal regions of the brain early in AD [9,163,164] providing this treatment upon initial cognitive decline may be critical to preserving cognitive function, as well as motivating clearance of pathology in the glymphatic system. This may not just be limited to gamma activity, either, as Khedr et al. [99] also reported tDCS reduced serum Aβ while Ladenabauer et al. [81] improved slow-wave band power through slow-oscillation tDCS during a daytime nap. Regarding the latter, slow-wave sleep has been associated with restorative cognitive benefits and the cleansing of metabolites in cerebrospinal fluid [165]. Accordingly, disrupted slow-wave sleep has been linked with increased Aβ levels [166], and further research into enhancing sleep patterns through TES and NIBS may provide other fruitful avenues to treating AD.

Longevity of cognitive effects, whether domain-specific or global, were mostly restricted to the active treatment period, with few studies having longer-lasting effects following stimulation. This was true regardless of the schedule of treatment or methodology of TES montages. This could be attributed to the limited neuromodulatory capabilities of TES, which may not be affecting regions of interest with enough energy or specificity to elicit greater effects, but also to the dysfunction of pathology-laden neurons not benefiting sufficiently from TES. However, improvements were able to be kept relatively consistent or with lower decrements in studies with lesser-frequency maintenance sessions extending beyond the more intensive short-term protocol followed in most studies. Indeed, research has found that LTP-like effects of TES may be most pronounced when there is overlap between the lingering effects of a past session and the application of new stimulation [167]. As such, because TES devices are relatively simple and inexpensive, they may serve well as supplementary maintenance to other intensive treatments when used at home under consistent patient compliance, as demonstrated in the 6-month study of Im et al. [96].

Overall, TES findings were highly heterogeneous, and several sham-controlled studies reported null, weak, or only trending effects despite using plausible cortical targets [80,84,85,86,87,94,97,108]. This variability likely reflects differences in disease stage, target selection, current density, online versus offline task pairing, sham design, baseline medication status, and the cognitive domains assessed. Clinically, the most consistent TES signals appear to involve global cognition and episodic or associative memory in early-stage or repeatedly treated cohorts, but the overall evidence remains mixed, and durability is usually modest without maintenance dosing.

## 5. Results of TMS Studies

### 5.1. DLPFC

A total of 18 studies applied rTMS to the left, right, or bilateral DLPFC. MT intensities ranged from 70 to 120%, with those at lower values between 70% and 80% being used in only iTBS protocols. Total numbers of pulses delivered ranged from 1200 to 4800 pulses per day, with pulses exceeding 3000 being given over the course of multiple sessions per day or across different regions of the DLPFC.

Three studies with aMCI patients employed rTMS over the l-DLPFC. Compared to sham, ten consecutive daily sessions of verum alpha (10 Hz) rTMS proved effective in enhancing performance in the Rivermead Behavioral Memory Test (RBMT) in a study by Drumond Marra et al. [115]. These effects persisted at a 30-day follow-up, though the authors noted that practice effects may have played a factor in this stability. Meanwhile, beta (20 Hz) rTMS significantly improved visuospatial reasoning quantified through the Raven Colored Progressive Matrices (RCPM) task in Bagattini et al. [116]. This was achieved following 20 sessions over 4 weeks of CT paired with rTMS, which showed additive effects of verum stimulation. Additionally, both the generalized gains in the RCPM and the add-on effects of rTMS on CT persisted 3 months later. The third study, Aghamoosa et al. [117], employed 3 days of iTBS, which led to a significantly improved composite cognitive score from the NIH Toolbox for the Assessment of Neurological Behavior and Function Cognition Battery, although this study lacked a control group for comparison.

Nine sham-controlled studies using rTMS over the l-DLPFC were conducted on AD patients. Four of these used beta rTMS and found significant improvements in at least one test of cognitive functions. This was the case for ADAS-Cog scores in Wu et al. [118], which benefited from 20 daily sessions over 4 weeks. Improved MMSE scores after 30 daily sessions split across 6 weeks were additionally reported in Li et al. [119], and these changes were significantly correlated with changes in plasticity. While ADAS-Cog scores returned to baseline at a 3-month follow-up, MMSE scores continued to be improved over baseline. Alongside the two other cognitive tests, MoCA scores were also improved in Tao et al. [120] following 30 daily sessions over 6 weeks. Improvements in serum Aβ levels were also observed as early as 3 weeks into the treatment cycle. In another beta rTMS study by Cotelli et al. [121], however, the only significant improvements made across a series of memory and cognitive domains in moderate AD patients were in the sentence comprehension portion of the Battery for Analysis of Aphasic Deficits. These effects manifested after 10 daily sessions over 2 weeks, and while an additional 2 weeks of stimulation did not provide additional increases in these benefits, these improvements remained stable after an additional 8 weeks transpired.

Another three studies using alpha rTMS also found strong results in AD patients. In Padala et al. [122], improvements in 3MS test scores after 20 daily sessions over 4 weeks were made, with the effects remaining clinically significant at an 8-week follow-up. Over the same time frame (20 daily sessions over 4 weeks), with the addition of the left lateral temporal lobe as a stimulation target, Zhang et al. [123] found improvements in ADAS-Cog scores following an hour of CT during stimulation. These improvements were significantly greater in the verum group compared to sham, and these effects remained stable after a 4-week follow-up. In a cohort with more severe AD cases, 60 sessions over 2 months were given in Zhang et al. [124] and resulted in significant improvements in the Severe Impairment Battery.

Lin et al. [125] found significant improvements in MoCA scores and across all six domains of an Auditory Verbal Learning Test (AVLT) following 14 consecutive days of twice-daily iTBS in verum AD patients, with no changes in the sham group. Beta activity within and the natural frequency of the l-DLPFC were both increased, as well. Another iTBS study, Wu et al. [126], also distributed stimulation over 14 consecutive days, though with three sessions given per day. Here, significant improvements in associative memory, general memory, attention, language, executive function, visual–spatial function, MoCA, and MMSE scores were reported in the verum group post-treatment and remained stable at an 8-week follow-up.

Only two studies targeted the r-DLPFC. Cotelli et al. [127] stimulated both sides individually as mild and severe AD cohorts were given beta or sham stimulation. Mild patients performed better at an action naming task compared to sham, and severe patients had additional improvements in object naming as well. There were no significant differences in stimulation of the different sides, though both performed better relative to sham. In aMCI patients, Cui et al. [128] found improvements after receiving 10 daily sessions of alpha rTMS over 2 weeks compared to sham. These were observed in the AVLT—Immediate free recall and 5 and 20 min delayed recall, with these improvements lasting through to a follow-up 8 weeks later. Additionally, AVLT—Recognition was trending towards significance after treatment and became significant at follow-up. Functional connectivity within the DMN was decreased following verum treatment, and lower resting-state functional connectivity within the DMN at baseline was more predictive of verum treatment efficacy, suggesting hyperconnectivity owed to the neurotoxic effects of AD-related pathology may influence the efficacy of rTMS across the progression of the disease. Studies applying sequential stimulation to both hemispheres of the DLPFC mostly used beta frequency stimulation. Ahmed et al. [129] compared 1 Hz to 20 Hz (and sham) stimulation frequencies in a mix of mild to moderate and severe AD patients. Compared to sham, improvements in MMSE scores were made after five consecutive verum sessions daily only in the 20 Hz cohort of mild to moderate patients, and these effects remained stable at 1- and 3-month follow-ups. In another study, Moussavi et al. [130] divided its subjects across three potential treatment schedules: 20 sessions of verum across 4 weeks, 10 sessions of verum across 2 weeks, or 20 sessions of sham across 4 weeks. Improvements were made across the three groups up to 6 months later, though no differences between verum or sham stimulation were observed. The researchers attributed this potentially to their use of a sham coil which produced a weak electrical field intended to give sensation, a tool that was also used in many studies contained within this review. Referencing another study by Opitz et al. [168], the researchers theorized that this weaker field from a sham coil remained capable of exciting underlying neurons, thus creating LTP-like effects even in the sham group. Another beta rTMS study by Rutherford et al. [131] featured an online component as subjects were asked to name objects in the 5 s interval between pulses. First, 10 daily sessions over 2 weeks of sham or verum stimulation were given, with 3 additional maintenance sessions over 2 subsequent weeks. Significant improvements in MoCA scores were found under verum stimulation after the second and third weeks of stimulation for both early- and late-stage AD patients, though the improvements in earlier-stage subjects were greater. While these improvements remained by the fourth week in both groups, they were no longer statistically significant. At the time of publication, six patients from the initial study had received 10 additional verum maintenance sessions over 2 weeks every 2 to 7 months over a total of 19 months. All patients had better than expected decrements in MoCA scores compared to existing longitudinal data, with two of them actually making further improvements. Four of the six additionally had better than expected decrements in ADAS-Cog scores. The one other bilateral DLPFC study, Zhou et al. [132], applied delta (1 Hz) and alpha frequency stimulation to the right and left DLPFC, respectively, in each session. After 20 daily sessions over 4 weeks, the verum group featured significant improvements in PSQI and ADAS-Cog scores, which remained stable at an 8-week follow-up.

### 5.2. NeuroAD Therapy

Similar to the Andrade et al. [110] protocol described in the TES section, multiple studies used a split session schedule where three brain regions including Broca’s area, Wernicke’s area, left or right DLPFC, and the left or right somatosensory association cortices (SACs) were stimulated per session. This technique (and the proprietary hardware and software since developed for it) has been dubbed NeuroAD therapy, which additionally includes CT components associated with the brain area being stimulated. Eight studies used NeuroAD therapy with intensities ranging from 90 to 110% rMT and total pulse numbers of 1200–1500 split between three brain areas.

Seven studies used alpha rTMS paired with NeuroAD therapy in mild to moderate AD patients. The ADAS-Cog and MMSE scores of early and moderate AD patients were improved in Bentwich et al. [133] after 30 daily sessions spread over 6 weeks. A total of 24 additional biweekly maintenance sessions were given after, and ADAS-Cog scores remained stable for another 3 months. Nguyen et al. [134] also found significant improvements in ADAS-Cog scores post-treatment (albeit with a shorter schedule of 25 sessions over 5 weeks). Additionally, this study also stimulated both hemispheres of the prefrontal cortex, for a total of four brain areas stimulated per session. Without additional maintenance sessions, the best responders remained improved at a 6-month follow-up while others returned to about baseline levels. Notably, half of this study’s cohort featured baseline memory impairment levels comparable to MCI, while the other half ranged from early to severe AD. While the former two studies did not have a randomized, sham-controlled component, Rabey et al. [135] and Lee et al. [136] found significant improvements in ADAS-Cog scores in their verum groups while sham groups worsened after 6 weeks of treatment. Rabey et al. [135] gave the same biweekly maintenance treatments as Bentwich et al. [133] and reported similar stability in ADAS-Cog scores after 3 months, but the improvements in Lee et al. [136] were further improved at a six-week follow-up with no additional treatment sessions. The latter study also found that mild AD patients made greater improvement relative to those with moderate AD under both verum and sham stimulation. Although the verum group showed significant within-group improvement from baseline and the sham group did not, the between-group difference at the end of stimulation was not significant; a similar pattern was reported in Sabbagh et al. [137]. Significant between-group differences in ADAS-Cog scores were, however, observed at a 6-week follow-up in Sabbagh et al. [137], and the verum group remained improved while the sham group decremented to baseline. In addition to having higher motor-threshold values, the researchers also noted that patients with baseline ADAS-Cog scores lower than 30 (indicating milder AD) made larger improvements in the instrument—as well as demonstrating better CT performance—after verum stimulation, whereas patients with higher baselines were more variable.

The above five studies each employed rTMS and CT with double respective placebos for their sham groups that did not test for additive effects of rTMS relative to CT. Brem et al. [138] elucidated this potential interaction factor using NeuroAD treatment in groups with CT paired with rTMS, CT paired with sham rTMS, and sham CT paired with sham rTMS. Both of the true CT conditions paired with verum or sham rTMS had significantly larger improvements in ADAS-Cog scores compared to sham training with sham rTMS, though there was no immediate add-on difference of rTMS paired with real CT. At follow-up sessions 4–6 weeks after treatment was finished, however, only those who received verum rTMS with real CT remained significantly improved compared to baseline, and further improvements were made compared to post-treatment in two-thirds of the patients. The researchers additionally found that the plasticity of patients’ brains was also predictive of the degree of clinical response they made to rTMS. Under the same groupings and treatment time frame, Vecchio et al. [139] did not find significant post-treatment improvements in the ADAS-Cog scores of either the CT/rTMS or the CT/sham groups, although both treated groups were significantly different from the sham/sham group, which had a decrement. At a 40-week follow-up, all groups presented with decrements, though the CT/rTMS group had the lowest with a return to a little above average baseline values (and was statistically lower than the other two groups), with the CT/sham group double that of the rTMS group, and the sham/sham’s average increasing by nearly five times that of the CT/sham group (albeit with a small final sample size of only two participants). Additionally, Vecchio et al. [139] observed that the small world index of delta, theta, alpha, beta, and gamma band activity, while similar post-treatment in the two CT groups, remained stable in the follow-up only in the CT/rTMS group, with significant differences across the frequencies observed in the CT/sham group.

One other study, Alcalá-Lozano et al. [140], used a NeuroAD-like treatment plan, albeit with theta (5 Hz) rTMS and no reported use of CT. Significant improvements in ADAS-Cog and MMSE scores were produced after 15 daily sessions of NeuroAD-like therapy over 3 weeks. These improvements persisted 4 weeks later, although the results at both time points were not significantly different from another group in which only the l-DLPFC was stimulated. Though it should not be considered superior to DLPFC stimulation, NeuroAD treatment should also not be considered equal to it, given these differences in stimulation and scheduling protocols.

### 5.3. Parietal Lobe

A total of four studies targeted the precuneus using beta (20 Hz) rTMS at 100% rMT with a total of 1600 pulses per session. In a cohort of aMCI patients, Koch et al. [141] reported significant improvements were made in the RAVLT delayed recall subsection (though not immediate recall) under verum stimulation when compared to sham after 10 daily sessions spread over 2 weeks. Additionally, significant increases in beta activity were observed in the precuneus following verum stimulation. In a mixed cohort of MCI-due-to-AD and mild AD dementia patients, after 20 sessions split over 4 weeks, Jung et al. [142] found ADAS-Cog scores were significantly improved in the verum group compared to sham. These remained significant and further improved 4 weeks later. Additionally, increased functional connectivity between the hippocampus and precuneus was measured in the verum group, and these improvements were correlated with the improvements made in ADAS-Cog scores. Another study by Koch et al. [143] applied 10 daily sessions over 2 weeks followed by 22 weekly maintenance sessions in a mild to moderate AD patient group. At the final week, ADAS-Cog and MMSE scores were significantly different between the verum and sham groups, with verum patients remaining stable and sham patients showing significant declines. These effects remained stable in the same cohort after an additional 38 maintenance sessions were given, making for a total of 52 weeks of stimulation as reported in Koch et al. [144]. At the 12th, 24th, 36th, and 52nd weeks of observation, verum patients had significantly higher ADAS-Cog scores compared to sham. At Week 52, the average worsening of sham subjects compared to verum subjects was nearly doubled in ADAS-Cog and more than tripled in MMSE. Although there was a reported increase in precuneus gamma activity at the 24th week in verum patients, there were no differences in EEG activity between verum and sham by the 52nd week.

Several studies targeted the angular gyrus. At 100% rMT, Chen et al. [145] applied 1600 pulses of beta rTMS to the left angular gyrus in both aMCI and AD patients over 20 daily sessions in 4 weeks. They observed significant improvements in MoCA scores from baseline to post-treatment in those who received verum stimulation, as well as significantly improved composite memory function scores. aMCI patients also significantly improved in a composite language function score. Additionally, the researchers found these improvements were able to be predicted by baseline default mode network and larger-scale network connectivity. In another study, Liu et al. [146] sequentially stimulated the left and right angular gyrus with 2400 gamma (40 Hz) rTMS pulses for each of its 12 daily sessions over 4 weeks in AD patients. Subjects who received verum stimulation featured increased gamma band power in the left temporoparietal cortex and made significant improvements in MMSE, MoCA, and ADAS-Cog scores from baseline to post-treatment, which remained stable and significantly improved from baseline at an 8-week follow-up. Widespread enhancements in functional connectivity were also observed in the verum group as connectivity increased between the bilateral angular gyrus while anterior and posterior regions of the brain were strengthened, and these changes in functional connectivity were correlated with performance in the three cognitive assessments.

In a bid to influence hippocampal activity in AD patients, two other studies identified personalized regions in the left parietal lobe with the highest functional connectivity to the hippocampus. Alpha rTMS at 100–110% rMT was applied in 10 daily sessions over 2 weeks in both studies. Jia et al. [147] found significantly greater improvements were made in verum patients on the 12-Word Philadelphia Verbal Learning Test (PVLT) total score and the MMSE when compared to sham. The immediate and short-delay recall subscales of the PVLT were also improved, as well as the orientation subscore of the MMSE in verum compared to sham. In the other study, Wei et al. [148], the custom target area was localized more specifically within the left lateral parietal lobule, but the results were similar. In their mild to moderate AD patients, the verum group showed improvement in MMSE and PVLT scores, although only the PVLT effects were significant relative to sham. Aside from the improved PVLT total scores, the immediate recall component was also significantly improved compared to sham. Both the MMSE and PVLT were not significantly different from sham at a 12-week follow-up in either group.

### 5.4. Other Brain Regions

Beta rTMS at an undisclosed rMT intensity was applied to the parietal and posterior temporal lobes (P3/P4 and T5/T6, respectively, on the 10–20 EEG system) of AD patients in Zhao et al. [149]. Three areas were targeted sequentially per session over a total of 30 sessions across 6 weeks, and CT tasks were delivered between stimulation of each brain area. Although no significant between-group differences were observed post-treatment or at follow-up, subjects who received verum stimulation showed significant within-group improvement from baseline in ADAS-Cog scores, which was further improved at follow-up. Sham patients, meanwhile, had non-significant changes at either time point. Interestingly, MMSE and World Health Organization University of California—Los Angeles AVLT scores were not significantly improved from baseline until the follow-up phase in the verum group, and MoCA scores came close to trending toward improvement at this time point, as well. When analyzed as mild or moderate AD cohorts, the researchers additionally observed that the milder AD patients were making greater gains from rTMS whereas moderate AD patients who received verum stimulation did not feature significant differences between time points in any cognitive assessment.

In a group of AD patients, Hoy et al. [150] used a unique multi-site stimulation protocol, targeting the l-DLPFC, r-DLPFC, left posterior parietal cortex (PPC), and r-PPC sequentially in order every session. Verum subjects showed significant within-group improvement from baseline to post-treatment in delayed recall, whereas sham subjects declined after a small improvement in Week 3. However, the between-group comparison on the assessment was not significant at post-treatment or follow-up, even though gamma network activity was significantly greater in the verum group by the end of 6 weeks. This network enhancement included frontal, parietal, and occipital regions, which altogether featured increased activity within and between hemispheres of the brain. Additionally, the researchers found that improvements in the episodic memory assessments were predictable both by baseline and post-treatment gamma connectivity.

In a mixed cohort of aMCI and early AD patients, Eliasova et al. [151] targeted the right inferior frontal gyrus with alpha rTMS at 90% rMT. Within a single session with 2250 pulses, significant improvements were made from baseline to post-treatment in Trail-Making Tasks (TMTs) A and B after verum stimulation, but not sham.

Yao et al. [152] applied 2000 pulses of 90% rMT theta rTMS to the bilateral cerebellum in 20 daily sessions over 4 weeks in AD patients. MMSE, MoCA, ADAS-Cog, RAVLT, Symbol Digit Modalities Test, BNT, Verbal Fluency Test, Clock-Drawing Task, and TMT-B scores were all significantly improved post-treatment in the verum group, with the effects remaining stable and still improved relative to baseline at an 8-week follow-up. In the verum group, functional connectivity was also increased between the bilateral cerebellum and three other brain regions: the DLPFC, the medial frontal cortex, and the cingulate cortex.

### 5.5. Discussion of TMS Studies

TMS in aMCI and AD patients improved global cognition and memory, as well as domains of associative memory, visuospatial reasoning, semantic memory, language and verbal memory, working memory, episodic memory, and executive function. Improvements in brain oscillatory power were also found in multiple studies [139,146] with another additionally finding reduced Aβ levels after 3 weeks [120]. While other hub regions such as the precuneus and angular gyrus also provided promising results that could benefit from future research, the DLPFC’s capacity as a network hub has garnered it the most attention in TMS research with the most variety of stimulation parameters. Whether it was isolated to the left hemisphere or bilateral, DLPFC TMS was effective in improving memory and cognition, often showing long-lasting effects extending up to 6 months later. Most DLPFC studies did not include online components, and while those that did include CT had beneficial effects, they did not tend to feature greater benefits in comparison. On average, studies using higher frequency 20 Hz rTMS with long-lasting effects had greater longevity than those using 10 Hz, though there was wide variability overall. While iTBS consistently produced improvements post-treatment, only one iTBS study produced long-lasting effects [126]. Granted, only one other study conducted follow-up assessments, but the differences in treatment schedules were notable; whereas Aghamoosa et al. [117] concentrated eight treatments into each session across 3 non-consecutive days, Wu et al. [126] included only three treatments across a greater span of 14 consecutive days. These findings could alternatively suggest that patients more progressed in the disease may benefit more from stimulation, given the populations of aMCI and AD patients in Aghamoosa et al. [117] and Wu et al. [126], respectively. Regardless, further research on iTBS may be valuable in establishing how its long-term effects may compare in protocols featuring different methods of consolidated delivery.

As noted in the TES section, CT was enough to elicit improvements in cognition and memory, although electrical stimulation did not provide additive effects. NeuroAD treatment, however, consistently improved global cognition in sham-controlled studies, suggesting that rTMS may provide substantial neuromodulatory benefits which further facilitate the mental stimulation created by CT. Interestingly, multiple NeuroAD studies did not find significant improvements until follow-up sessions as far as 40 weeks after treatment, which may suggest that the greatest impacts of rTMS may be delayed through LTP-like effects. NeuroAD studies had relatively homogenous protocols with 10 Hz frequencies and treatment schedules ranging from 25 to 30 sessions, and given the consistent effects produced across studies, may cement it as a highly effective treatment for aMCI and AD. Further research employing other stimulation parameters such as iTBS may elucidate still more effective treatments.

Other regions of interest improved cognition and memory, though two notable protocols produced powerful, widespread effects, despite being contained to single, sham-controlled studies. Despite a relatively low rMT of 40%, 40 Hz sequential stimulation to the left and right angular gyrus markedly increased functional connectivity and gamma activity across the left temporoparietal cortex, which was associated with improvement in three different assessments of global cognition [146], a rare finding. In contrast to the longevity contained to active treatment in tACS studies and another angular gyrus study using 20 Hz stimulation [145], these effects persisted 8 weeks later, and given the benefits of temporal gamma activity described in the TES section, further research into gamma band promotion may yield other efficacious treatment solutions. Another notable study found bilateral cerebellum stimulation with 5 Hz rTMS, too, improved three different assessments of global cognition and a multitude of memory domains with 8 weeks of post-treatment stability [152]. The wide diffusion of enhanced functional connectivity to the DLPFC, medial frontal cortex, and cingulate cortex demonstrated in this study could make the cerebellum a promising target for future NIBS research.

Compared with TES, the TMS literature contained a larger proportion of multi-session sham-controlled studies and more frequent follow-up assessments, which likely contributes to the impression of greater efficacy and durability. Even so, important sources of heterogeneity remain, including disease stage, target selection, stimulation frequency, motor-threshold scaling, total pulse dose, maintenance schedules, and whether treatment was paired with cognitive training. Across studies, more durable benefit was most often observed with multi-week protocols, high-frequency stimulation, and repeated or maintenance sessions, whereas single-session or shorter protocols more commonly produced immediate or domain-limited effects. These trends should be interpreted cautiously, however, because direct dose–response comparisons across protocols remain scarce.

## 6. Review of tFUS Studies

### 6.1. tFUS Studies

The first proof-of-concept study informing current tFUS research on cognitively impaired populations was run by Beisteiner et al. [153], in which three sessions per week over a total of 2–4 weeks were given in AD patients. During every session, a total of 6000 pulses with a PRF of 5 Hz and EFD of 0.2 mJ/mm^−2^ were sequentially delivered across the bilateral frontal cortices (800 per hemisphere, twice), bilateral lateral parietal cortices (400 per hemisphere, twice), and the extended precuneus cortex (600 at one bilateral target, twice) at one research site, while a second site distributed the same number of pulses across the whole scalp using a non-navigated global stimulation approach. The Consortium to Establish a Registry for Alzheimer’s Disease (CERAD) total scores and logistic regression scores were significantly improved post-treatment and at 1- and 3-month follow-ups compared to baseline, and principal component analyses further revealed that memory and verbal abilities continuously improved across each time point. Differences were observed between the research centers owing to the different protocols, though these were considered small. Regardless, functional connectivity was significantly increased in the memory network including the hippocampus, parahippocampal cortex, parietal cortex, and the precuneus, and these effects were significantly correlated with the behavioral improvements made. In a subsequent analysis of the center 1 patients, correlations revealed behavioral improvements were significantly associated with thickness in the left precuneus and left superior parietal lobule [154]. Further analysis by Dörl et al. [155] found untreated brain areas in the visuo-constructive network additionally featured less inter-network connectivity, which was associated with decrements in the figural component of the CERAD. While initially only trending post-treatment, these correlations were significant by the 3-month follow-up.

Targeting the same brain regions under the same stimulation parameters as center 1 of Beisteiner et al. [153], Matt et al. [156] included a sham-controlled component in a cohort of aMCI and AD patients. A PRF of 5 Hz was used, with a DC of 0.0015% and intensity of 24 mW/cm^2^ with 6000 total pulses. Here, each subject received six daily sessions spread over 2 weeks under sham and verum stimulation, with a 5-week washout in between conditions, though due to what appeared to be a carryover effect from the first part of the study going into the second condition, only significant effects were found within that first branch. Although analyses did not differentiate between aMCI and AD, interactions were found when the subject pool was split based on age. In the group of subjects aged 70 or younger, significant interactions between time point and stimulation condition were observed, with those who received verum treatment featuring higher CERAD total scores compared to sham at post-treatment and 1- and 3-month follow-ups. While the sham group stayed fairly similar, the verum group made large improvements post-treatment that stayed stable or further improved at the two follow-up sessions. In the group of subjects older than 70, no significant group differences were observed, although improvements were made across each time point. Across both age groups, subjects who received verum stimulation featured significantly higher post-treatment activity in the precuneus, visual areas, and the superior and inferior frontal gyri compared to sham.

Another study by Cont et al. [157] in mild to severe AD patients included the bilateral temporal cortices in their list of brain targets, otherwise following in the vein of Matt et al. [156] and Beisteiner et al. [153]. At a PRF of 4 Hz and with an EFD of 0.20 mJ/mm^2^, treatment was given as 6000 pulses every 2 days over 2 weeks for a total of 6 sessions, or 3000 pulses every day over 12 sessions. Behavioral results were not distinguished between the different protocols, but significant improvements in ADAS-Cog scores were observed at the end of treatment relative to baseline. Also using a PRF of 4 Hz but with an EFD of 0.25 mJ/mm^2^, Shinzato et al. [158] targeted frontotemporal, parietal, and occipital regions twice a week for 5 consecutive weeks. Improvements in ADAS-Cog scores were not significant post-treatment, but trending improvements were observed at a 90-day follow-up, potentially suggesting delayed LTP-like effects of the protocol.

One other sham-controlled study by Shimokawa et al. [159] followed a full brain stimulation protocol under low-intensity pulsed ultrasound using convex transducers originating at the bilateral temporal bones. An FF of 0.5 MHz with a PRF of 781 Hz and DC of 5% at an intensity of 1.3 MPa was used in a cohort of aMCI and mild AD patients, although analyses distinguishing the clinical groups were not conducted. Treatment was given every other day for three days a week once every 3 months over a total of 18 months. No significant differences in the Japanese ADAS-Cog were observed between groups after 72 weeks, although verum subjects tended to have stable scores whereas the sham group saw gradual (though non-significant) decrements over time.

One other study targeted isolated brain areas. Jeong et al. [160] used an FF of 250 kHz and a PRF of 2 Hz with 4% DC and a SD of 300 ms at an intensity of 3 W/cm^2^. The right hippocampus was targeted in a group of moderate to severe AD patients, and after one session, immediate recall and recognition memory in the Seoul Verbal Learning Test (SVLT) were significantly improved. These were accompanied by and correlated with increased glucose metabolism in the right hippocampus.

### 6.2. Discussion of tFUS Studies

The field of tFUS is still in its early stages, especially in terms of working with cognitively impaired populations. Although the available studies suggest that frontal, parietal, precuneus, and hippocampal tFUS targets can influence global cognition or memory [153,156,158,160], the evidence base remains much smaller than that for TES or TMS and includes relatively few randomized, sham-controlled clinical trials. Consequently, any clinical interpretation must remain cautious. At present, tFUS is better viewed as a promising emerging modality than as an established therapeutic option for aMCI or AD. Its major translational appeal lies in its capacity for comparatively focal stimulation and access to deeper structures such as the hippocampus, which are highly relevant to dementia. However, these same advantages underscore the need for more careful dose-finding, long-term safety monitoring, target-validation work, and biomarker-rich studies that can determine whether short-term cognitive effects correspond to durable neurobiological change.

The current human safety literature is encouraging but still limited, and the comparative clinical translation of tFUS therefore remains premature relative to TES and TMS (Table 4). From a comparative safety perspective, TES generally produces the mildest adverse-effect profile, most commonly scalp sensations, itching, tingling, or headache. TMS is also usually well tolerated but carries a greater burden of scalp discomfort and the rare but important risk of seizure under inappropriate dosing. TFUS has thus far produced mainly mild transient complaints in small samples but lacks the long-term safety database available for TES and TMS. For vulnerable older adults, tolerability, treatment burden, and the need for repeated sessions are therefore central clinical considerations alongside efficacy.

**Table 4:** Cross-modality synthesis of major features of the current clinical evidence base.

Modality	Typical Evidence Base	Common Targets/Designs	Most Consistent Cognitive Signals	Durability Pattern	Safety/Translational Notes
TES	Many small sham-controlled, crossover, or repeated-session studies; some open-label components	DLPFC, temporal cortex, precuneus, angular gyrus; often paired with CT or WMT	Global cognition; episodic, associative, and working-memory outcomes in selected cohorts	Often limited to the treatment period unless repeated or maintenance dosing is used	Generally mild scalp discomfort or headache; accessible and practical for home-based adjunctive use
TMS	Larger multi-session clinical literature with more sham-controlled studies and follow-up assessments	DLPFC, NeuroAD multi-site protocols, precuneus, angular gyrus, cerebellum	Global cognition, episodic/associative memory, language, executive function	Most durable signals in multi-week, high-frequency, or maintenance-session protocols	Usually well tolerated; scalp discomfort and rare seizure risk require tighter dosing and supervision
tFUS	Small emerging literature with relatively few randomized sham-controlled studies	Frontal and parietal cortex, precuneus, whole-brain protocols, hippocampus	Preliminary signals for global cognition and hippocampal-dependent memory	Insufficient evidence to confidently define durability	Promising focality and deeper targeting, but clinical translation and long-term safety remain premature

Note. TES = transcranial electrical stimulation, TMS = transcranial magnetic stimulation, tFUS = transcranial focused ultrasound stimulation, DLPFC = dorsolateral prefrontal cortex, CT = cognitive training, WMT = working memory training.

## 7. Limitations

In describing and comparing NIBS research in aMCI and AD, this review was intended as a broad synthesis rather than a formal meta-analysis. Several limitations therefore constrain interpretation. First, the search strategy relied on PubMed plus backward citation searching rather than multiple bibliographic databases. Although PubMed captures a large proportion of the high-quality relevant biomedical and clinical literature, this choice may have reduced study coverage and may have omitted some eligible reports indexed elsewhere. Second, substantial heterogeneity across sample size, disease stage, medication status, diagnostic confirmation, stimulation parameters, sham conditions, outcome measures, and follow-up intervals limits direct comparison across studies. Third, this review did not apply a formal risk-of-bias instrument. That decision reflected the descriptive and cross-modality scope of the review and the marked heterogeneity of the included studies, but it also means that the strength of evidence should be interpreted cautiously because the literature includes a mixture of randomized sham-controlled trials, crossover designs, and open-label studies with uneven methodological rigor.

In addition, the clinical literature still provides limited biological validation of treatment effects. Behavioral improvement does not necessarily imply disease modification, and relatively few studies integrated neuroimaging, electrophysiology, fluid biomarkers, or pathology-linked biological endpoints that could clarify whether NIBS altered core disease processes. This translational gap is especially important in light of preclinical work suggesting that neuromodulation may influence neurotrophic signaling, neuroinflammatory cascades, oxidative stress, mitochondrial function, insulin signaling, and related mechanisms relevant to AD pathophysiology [12,13,19,22,24,27]. More specifically, future translational studies would benefit from linking clinical outcomes to biological validation endpoints such as amyloid- or tau-related markers, inflammatory or neurotrophic measures, network-level imaging changes, and—where preclinical work is concerned—protein-expression assays, Western blot and enzyme-linked immunosorbent assay (ELISA) measures, histology or immunohistochemistry, and validated memory paradigms in animal models. Future work would also benefit from standardized dose–response studies, clearer therapeutic-window definitions, and longer safety follow-up.

A final limitation is that the NIBS literature is expanding rapidly, and some emerging protocols with possible clinical relevance are not yet fully represented in the peer-reviewed record. One example is the “F10” tDCS protocol, which has shown large effects on learning in healthy younger adults [169,170] and has generated encouraging preliminary findings in healthy older adults and up to 10-fold improvement in learning of a difficult discovery learning task in an aMCI group [171]. Because this and other new approaches have not yet been evaluated extensively in fully published peer-reviewed clinical studies, their relevance for treatment remains provisional, but promising.

## 8. Conclusions

As neurodegenerative illnesses such as MCI and AD become more prevalent in elderly populations, finding effective solutions to halt or slow progression of these diseases is critical to ensuring adequate quality of life. Across the studies reviewed, the most reproducible cognitive signals involved global cognition, episodic or associative memory, and selected executive functions, particularly when stimulation targeted network hubs and was delivered in multi-session protocols. TES may be attractive as an accessible adjunct or maintenance intervention, but its effects were often modest or short-lived without repeated dosing. TMS presently has the strongest clinical evidence for more durable benefit, especially in multi-session, sham-controlled studies and network-guided protocols. tFUS remains promising because of its focality and ability to focus on deeper structures, but its clinical evidence base is still preliminary. Overall, early intervention, precision targeting, standardized study design, and incorporation of biomarkers with longer-term follow-up will be central to determining the ultimate therapeutic role of NIBS in dementia. In particular, future progress will depend on studies that pair cognitive outcomes with stronger biological validation of target engagement and disease-relevant mechanisms.

## Data Availability

No new data were created or analyzed in this study.
